# Activated T-cell membrane-derived nanocargoes displaying multi-immune checkpoints for enhanced cancer immunotherapy

**DOI:** 10.1016/j.mtbio.2025.102702

**Published:** 2025-12-24

**Authors:** Li Du, Xiaoying Zhang, Yao Gong, Miaoshu Liu, Jide Sun, Xingping Hu, Jian Peng, Zhangling Liu, Ting Zhang, Jie Xu, Fengxia Gao, Wei Cheng

**Affiliations:** a The Center for Clinical Molecular Medical Detection, Innovative and Translational Laboratory of Molecular Diagnostics, Laboratory Medicine Center, The First Affiliated Hospital of Chongqing Medical University, Chongqing, 400016, China; b Biobank, The First Affiliated Hospital of Chongqing Medical University, Chongqing, 400016, China; c Department of Laboratory Medicine, The First Affiliated Hospital of Chongqing Medical University, Chongqing, 400016, China; d Western Institute of Digital-Intelligent Medicine, Chongqing, 401329, China

**Keywords:** Activated, T-cell membrane vesicles, Multi-immune checkpoints, Immunotherapy

## Abstract

The advent of immune checkpoint inhibitors (ICIs) has significantly transformed the landscape of cancer treatment in the last decade. However, the efficacy of single-agent ICI remains constrained due to multiple immune checkpoints (ICs)-mediated T cell suppression and inadequate T cell tumor infiltration. Here, we developed a novel approach using activated T-cell membrane-guided nanocarriers to simultaneously block multiple ICs and enhance T-cell infiltration. Initially, primary T cell activation was induced in vitro, and T cells with high expression of ICs were selected to prepare T-cell membrane vesicles. These vesicles were then utilized to coat immunogenic inducer-loaded liposomes (dLNPs) to create nanocarriers termed AM-dLNPs. The AM-dLNPs were demonstrated to effectively inhibit multiple ICs pathways through competitive blockade of immune checkpoint ligand-receptor interactions and down-regulation of immune checkpoint ligand expression. Additionally, the AM-dLNPs exhibited a strong ability to promote intratumoral T cell infiltration through targeted delivery of the immunogenic inducer. Benefiting from the exceptional biosafety profile, multi-ICs blockade efficacy, and tumor-targeting properties of the T-cell membrane vesicles, administration of AM-dLNPs resulted in a significant reduction in tumor progression and notable survival advantages in various mouse tumor models. These findings provide a basis for the clinical assessment of activated T-cell membrane-derived nanocarriers, which solves the dilemma of limited effects of ICI treatment without biosafety concerns. Its versatility also enables the targeted delivery of other immunogenic agents for synergistic antitumor immunotherapies.

## Highlights

• STING agonist-mediated anti-tumor effects require synergistic multi-checkpoint blockade.
• Activated T-cell membrane vesicles possess multi-checkpoint blocking ability.
• Activated T-cell membrane-coated nanocargoes precisely deliver STING agonist to enhance tumor immunogenicity.
• Activated T-cell membrane-coated nanocargoes boost immunogenicity and block checkpoints for potent anti-tumor efficacy.

## Graphical abstract

**Figure:**
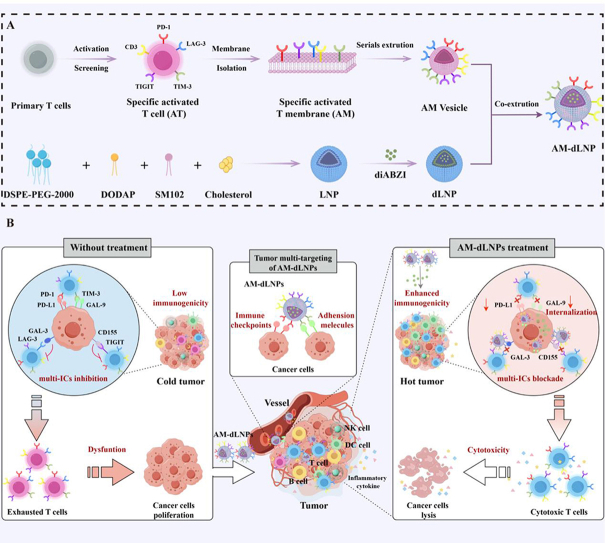

## 1 Introduction

Cancer immunotherapy has brought great hope to fight against cancer in recent years. Given the key role of T cells in antitumor immunity, immunotherapies have primarily focused on enhanced T cell activation and increased antitumor cytotoxicity [[1], [2], [3]]. Immune checkpoint inhibitors (ICIs) are the current mainstays of antitumor immunotherapies by blocking immune checkpoints (ICs), thereby restoring the antitumor ability of T cells [4,5]. However, the clinical response rates of available ICIs were still relatively low due to poorly tumor immunogenicity and complex immunosuppressive factors [6].

A key factor contributing to initial resistance to ICIs is the low immunogenicity of tumors and limited T cell infiltration, defining “cold tumors” [7]. To augment the efficacy of ICIs, combination strategies have been utilized to transform cold tumors into hot tumors. These approaches include combining PD-1/PD-L1 blockade with radiotherapy and chemotherapy, or with immunogenic inducer therapy [8]. However, chemoradiotherapy and immunogenic inducer therapy have been demonstrated to elevate certain immunosuppressive elements within tumors, including various ICs [9]. This suggests that heightened tumor immunogenicity correlates with increased expression of multiple ICs.

Due to the simultaneous presence of multiple ICs and their ligands within TME, depending on a single ICI to unleash the inhibition of anti-tumor T cell response proves inadequate [10,11]. Consequently, the exploration of multiple immune checkpoint blockades (ICB) for cancer therapy has been advocated. the clinical advancement of agents for ICB predominantly centers on ICs-targeted antibodies, with diverse antibody combinations being investigated for treating various cancers [12,13]. Nevertheless, unrealistic expectations, suboptimal treatment efficacy, and undesirable immune-related adverse events (irAE) impede research progress and restrict the application of such combination therapies [14,15]. Concurrently, mounting evidence underscores the necessity for simultaneous inhibition of immune checkpoint ligand-receptor interactions and downregulation of these molecules' expression to effectively alleviate T cell inhibition [16,17]. However, the majority of ICs-targeted antibodies are limited to competitively obstructing ligand-receptor binding without reducing the expression of these molecules. Therefore, the development of safe and effective multi-ICs blockade therapy is urgently needed.

Recent evidence has demonstrated that activating the cyclic GMP-AMP synthase-stimulator of interferon genes (cGAS-STING) signaling pathway is a powerful strategy for the conversion of immunologically 'cold' tumors to 'hot' ones, leading to a significant augmentation of anti-tumor immunity [18]. The cGAS-STING pathway, a cornerstone of innate immunity, senses cytosolic dsDNA and transduces this signal through the synthesis of cGAMP, leading to STING-dependent activation of the TBK1-IRF3 axis. This cascade drives type I interferon (IFN-I) production and promotes the recruitment and activation of cytotoxic T cells [19]. Concurrently, STING pathway activation in tumor cells activate antigen-presenting cells and promotes the infiltration of natural killer (NK) cells, culminating in a coordinated antitumor immune attack [20,21]. Several STING agonists, including cyclic dinucleotide analogs, metal ions, and small-molecule compounds, have advanced into clinical trials [22]. Innovative nanoplatform technologies have achieved potent suppression of both primary and metastatic tumors by synergizing conventional anticancer agents with self-amplifying cGAS-STING activation [23]. Furthermore, combination therapies co-administering STING agonists with immune checkpoint blockers, such as PD-1/PD-L1 inhibitors, exhibit promising synergistic anti-tumor effects [24]. While the combination of ICB with STING agonists represents a promising anti-tumor strategy, systemic administration of STING agonists may cause unintended inflammation or autoimmunity since these agonists are not specifically targeted to tumor sites [25,26]. Therefore, innovative strategies are required to sensitize tumors to STING agonists while minimizing irAE.

Given the potential of using a patient's own T cells for cancer treatment after amplification and modification, and noting that T cells can transiently and highly express multiple ICs upon activation [27,28], we hypothesized that activated T-cell membrane vesicles with high ICs expression could potentially disrupt multiple immune checkpoint inhibitions by competitively binding to and internalizing ICLs. Additionally, these vesicles were envisioned to function as carriers targeting tumors for STING agonist. Here, we first induced primary T-cell activation in vitro to screen activated T cells with high ICs expression, which were further produced into T-cell membrane vesicles. Subsequently, the activated T-cell membrane vesicles (AM) were engineered to encapsulate diABZI (a STING agonist)-loaded liposome for final nanocargoes assembly (AM-dLNPs) (Scheme A). We showed experimentally that AM-dLNPs achieved tumor multi-targeted delivery via membrane-originated ICs. Targeted delivery of diABZI enhanced tumor immunogenicity and intratumoral T cell infiltration. Furthermore, AM-dLNPs inhibited multiple ICs pathways by competitively binding to and endocytosing ICLs, thereby restoring T cell antitumor cytotoxicity (Scheme B). Benefiting from these synergic effects, AM-dLNPs induced efficient and safe antitumor immune responses, which led to significant reduction in tumor progression and marked survival benefits in multiple mice tumor models. Thus, strategies that enable pleiotropic activation of anti-tumor immunity with AM-derived nanocargoes would greatly facilitate its clinical translation for treating human cancers, particularly in settings where single immunotherapy is not effective.

**Scheme:**
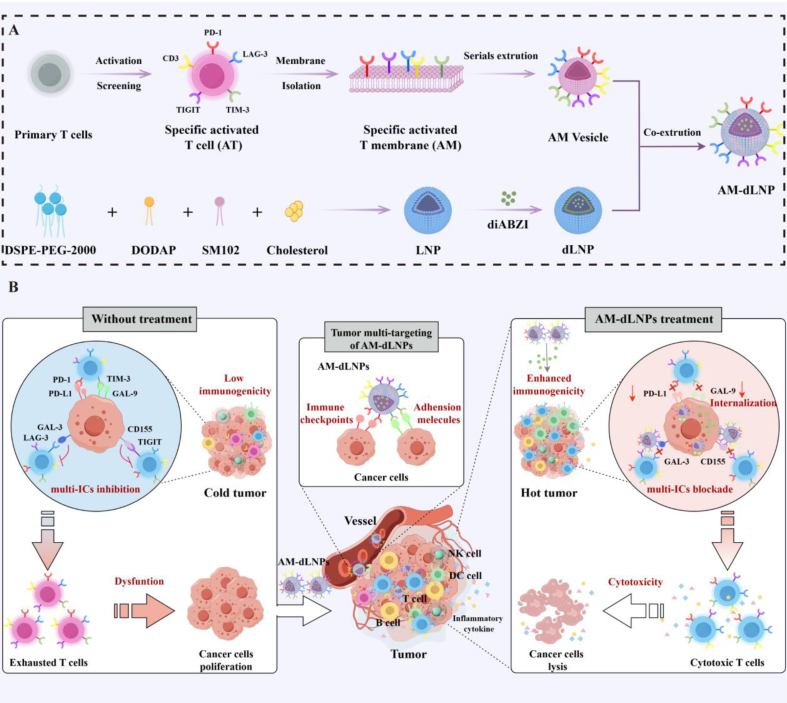
Schematic illustration of the main synthesis procedures and antitumor mechanism of AM-dLNPs.(A) Preparation of AM and immunogenic inducer-loaded AM-dLNPs. (B) AM-dLNPs enhance tumor immunogenicity and intratumoral T cell infiltration via AM-mediated tumor-targeted delivery of immunogenic inducer. AM-dLNPs block multi-ICs inhibition pathways by binding and internalization of cancer surface immune checkpoint ligands.

## 2 Results and discussion

### 2.1 High tumor immunogenicity accompanied by high expression of multiple ICs

ICI treatments are only effective in approximately 20 %–30 % of cancer patients whose tumors are generally hot tumors with high tumor immunogenicity [29]. One important reason is high tumor immunogenicity accompanied by high expression of multiple ICs, leading to the limited efficacy of single ICI therapy. The STING pathway is an indicator of a “hot” or immune-inflamed tumor, which plays an essential role in regulating the immunogenicity of tumor cells [30]. Accordingly, evidence suggested that high expression of STING in tumor tissues may be indicative of potent tumor immunogenicity. Meanwhile, some studies revealed that the high expression of STING in tumor tissue is directly associated with the expression of co-inhibitory ICs [31]. Here, we examined STING and ICs/ICLs expression in clinical tumor specimens, including PD-1/PD-L1, T cell immunoglobulin and mucin domain-containing protein 3 (TIM-3)/Galectin-9 (GAL-9), Lymphocyte activation gene-3 (LAG-3)/Galectin-3 (GAL-3). The results indicated a correlation between STING expression in cancerous tissues and the expression of ICs/ICLs. Tumors with elevated STING levels demonstrated increased expression of multiple ICs/ICLs (Fig. A-H).

**Fig. 1:**
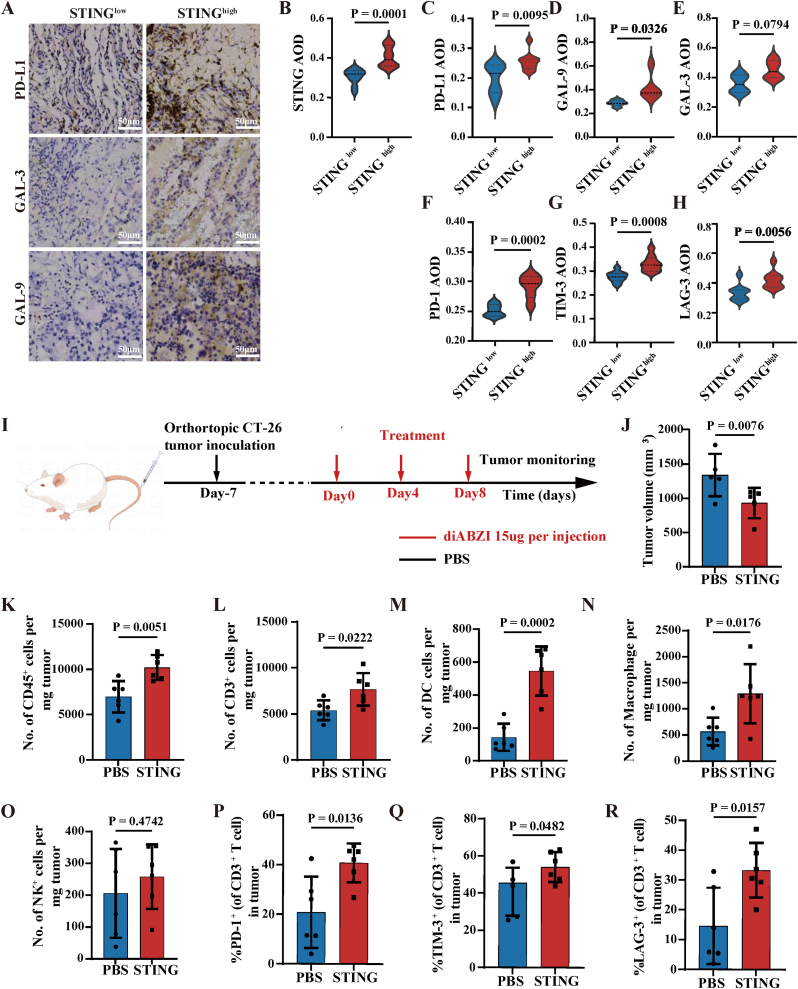
Association between intratumoural STING signature and ICs expression. (A–H) Representative IHC image and quantification of the expression level of STING and ICs including PD-L1/PD-1, GAL-9/TIM-3 and GAL-3/LAG-3 in lung cancer patients. (I) Experimental timeline and indicated treatment groups (three intravenous injections, diABZI: 0.75 mg per kg (body weight), n = 6 mice). (J) Statistical analysis tumor volume on day 13 of mice with CT-26 tumors receiving the indicated treatments. (K–O) Representative flow cytometric densities of total immune cells (CD45^+^), T lymphocytes (CD3^+^), DCs (CD11c^+^MHC-II^+^), Macrophages (CD11b^+^F4/80^+^) and NK cells (CD3^−^CD49b^+^) in the tumors receiving diABZI or PBS at three days after the last injections (n = 6 mice). (P–R) Representative flow cytometric quantification of the expression level of PD-1, TIM-3 and LAG-3 in tumor-infiltrating CD3^+^ T cells of CT-26-bearing mice receiving diABZI or PBS at three days after the last injections (n = 6 mice). All data are presented as the mean ± S.D. The *p* values were determined by two-tailed Student's *t*-test for (B–H) and (J–R).

ICI therapy is largely ineffective for the vast majority of immunologically cold tumors that have low immunogenicity and lack sufficient levels of tumor-infiltrating lymphocytes. Combination therapy has been used to turn cold tumors into hot tumors in order to increase the efficacy of ICIs in recent years. Here, we applied STING agonist to enhance the immunogenicity of tumors and examined whether the enhancement of tumor immunogenicity leads to upregulation of co-inhibitory ICs (Fig. 1I). Mice experiments showed diABZI treatment indeed upregulated multiple ICs expression, accompanied by enhanced infiltration of T cells, natural killer (NK) cells, dendritic cells (DCs), and macrophages in diABZI-treated tumors (Fig. 1K–R). Overall, experimental results demonstrated that activation of the STING pathway exhibited an anti-tumor effect while upregulating the expression of ICs/ICLs in tumor tissue (Fig. 1J). The limited efficacy of STING agonist-based therapy might be accounted for its-induced immune evasion by upregulating multiple ICs inhibition. The above clinical sample analysis and experimental mouse models suggest that tumors with high immunogenicity exhibit elevated expression of multiple ICs. Therefore, enhancing tumor immunogenicity in combination with blocking multiple ICs inhibition holds significant promise for improving cancer treatment outcomes.

### 2.2 Screening of activated T cells with high ICs expression

**Fig. 2:**
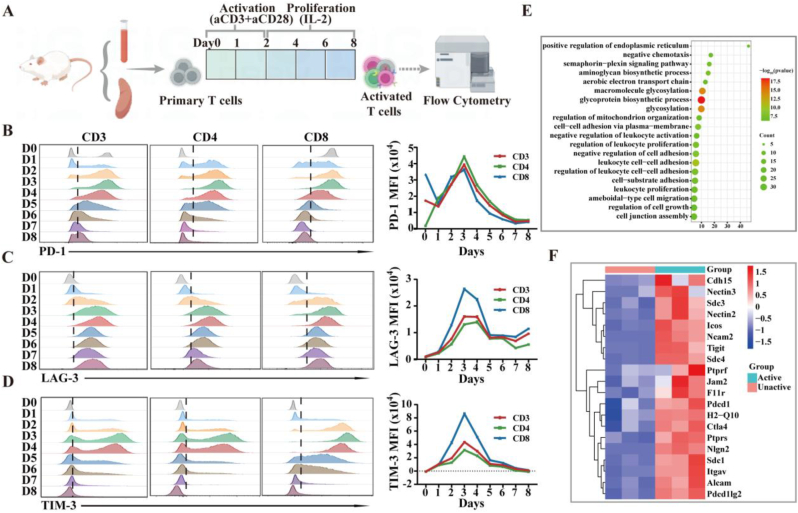
Screening activated T-cell with high expression of ICs. (A) Diagram of activated T cells with multiple ICs screening. (B–D) Representative flow cytometric histograms (left) and curves (right) of the expression level of ICs including PD-1 (B), LAG-3 (C) and TIM-3 (D) of activated T cells from day 0–8 of the T cell screening assay, gated on CD3^+^ T cells, CD4^+^ T cells, CD8^+^ T cells (n = 3 independent experiments), MFI, mean fluorescence intensity. (E) The KEGG pathway enrichment analysis of all types of membrane-associated genes that are upregulated in activated T cells compared with un-activated T cells. (F) Heatmap showing the scaled expression of membrane-associated genes in activated T cells compared with un-activated T cells. All data are presented as the mean ± s.d.

Prior research has demonstrated that cell membrane vesicles expressing PD-1 could inhibit the PD-1/PD-L1 pathway by competitively blocking the binding of PD-1 to PD-L1 and reducing PD-L1 expression [32]. This suggests that immune checkpoint molecules can be employed to impede the interaction of ICs on T cells with ICLs. Studies have indicated that activated T cells transiently and highly express multiple ICs, which, upon engagement with their ligands, hinder T cell activity to sustain immune tolerance [27]. We speculated that activated T lymphocyte membrane vesicles with rich ICs expression could serve as a broad-spectrum inhibitor of multiple ICLs through the mechanism of multivalent ligation-induced binding and internalization. To generate T cells with high ICs expression, we isolated primary T cells from mouse spleen or peripheral blood and induced activation in vitro. Subsequently, we assessed ICs expression at different activation stages (Fig. 2A). Our findings revealed peak PD-1, LAG-3 and TIM-3 expression on day 3 of T cell activation for both CD8^+^ and CD4^+^ T cells, which then declined to baseline levels over time (Fig. 2B–D). It is well known that exhausted T cells also express multiple ICs, but our further experiments showed that the activated T cells with high ICs expression were not exhausted T cells. The heightened ICs expression on day 3 was consistent across T-cell subsets (naïve, memory, and effector), as depicted in Fig. S1. Similar cyclic variations in ICs expression were noted in both naive and memory T cells. It indicated that the T cells on day 3 of activation have specific gene and protein expression characteristics.

Next, transcriptome sequencing was conducted to examine changes in gene expression profile in T cells after 3 days of activation. As the results showed in Fig. S2A, 732 membrane-related genes were found with significantly different expression changes (fold change >2, *P* value < 0.05) in total, of which 403 genes were down-regulated and 329 genes were up-regulated in specific activated T cells. KEGG analysis of the upregulated membrane-related genes revealed that these genes were mainly enriched in glycoprotein biosynthesis process, which was widely reported to regulate the expression of membrane protein receptors and adhesion molecules (Fig. 2E and F). To further prove the exact process, Gene ontology classification analysis was conducted to enrich these upregulated genes into function pathways. Among them, cell receptor interactions and cell adhesion pathways were enriched in the function pathways (Fig. S2B, Supporting Information). Both gene ontology and KEGG enrichment analyses illustrated that the expression of cell receptors and adhesion related genes was significantly different in activated and non-activated T cells, which are just essential in biomimetic cell membrane-based receptor blockers and nanocarriers.

### 2.3 Synthesis and characterization of AM-dLNPs

**Fig. 3:**
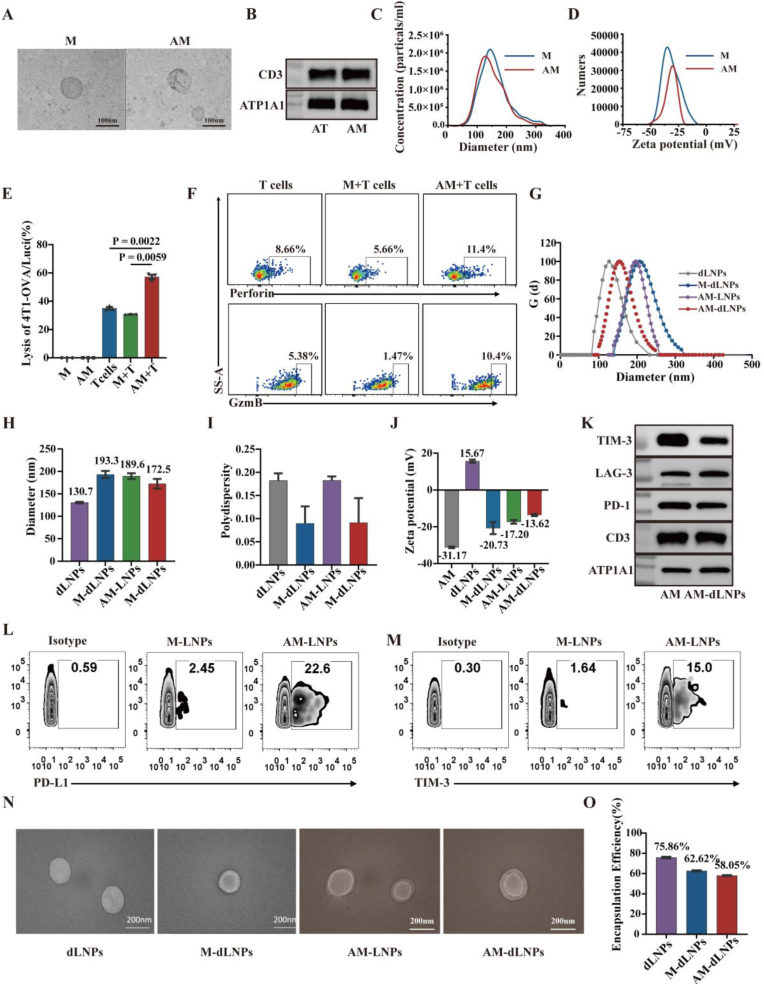
The preparation and characterizations of AM-dLNPs. (A)Transmission electron microscopy images of non-activated T cells membrane vesicles (M) and activated T cells membrane vesicles (AM). Scale bars, 100 nm. (B) Western blot analysis of CD3 protein in activated T cell (AT) and AM. (C, D) The average hydrodynamic diameter and surface zeta potential of AM and M as measured by dynamic light scattering (n = 3 independent experiments). (E) Rescue of ICs-mediated T-cell exhaustion by AM in cocultures of IFN-γ-pretreated (20 ng/ml, 24 h) 4T1-OVA/Luci cells and OT-1 T cells at an effector: target ratio of 5:1. 4T1-OVA/Luci cells death in the cocultures was evaluated by luciferase reporter assay system (n = 3 independent experiments). (F) Representative flow cytometric percentage of IFN-γ+, Perforin^+^ and Granzyme B^+^ (CzmB) in OT-1 T cells after co-incubation with IFN-γ-pretreated 4T1-OVA/Luci cells and diABZI, dLNPs, M-dLNPs, AM-LNPs, AM-dLNPs or PBS for 24h at an effector: target ratio of 5:1. (G–J) The average hydrodynamic diameter (G, H), polydispersity (I) and surface zeta potential (J) of T-cell membrane (TM), dLNPs, M-dLNPs, AM-LNPs and AM-dLNPs, as measured by dynamic light scattering (n = 3 independent experiments). (K) Western blot analysis of CD3, PD-1, LAG-3, and TIM-3 in dLNPs, M-dLNPs, AM-LNPs and AM-dLNPs. (L) Expression of PD-L1 on AM-derived nanocargoes was determined by Flow NanoAnalyzer. (M) Expression of TIM-3 on AM-derived nanocargoes was determined by Flow NanoAnalyzer. (N) Transmission electron microscopy images of dLNPs, M-dLNPs, AM-LNPs and AM-dLNPs. Scale bars, 200 nm. (O) High-performance liquid chromatography analysis of diABZI concentrations in dLNPs, M-dLNPs, AM-LNPs and AM-dLNPs (n = 3 independent experiments). dLNPs: diABZI-loaded liposomes, M-dLNPs: non-actived T-cell membrane-decorated diABZI-loaded liposomes, AM-LNPs: actived T-cell membrane-decorated empty liposomes, AM-dLNPs: actived T-cell membrane-decorated diABZI-loaded liposomes. All the data are presented as the mean ± S.D. The *p* values were determined by one-way ANOVA with Tukey's post-test for (E).

Next, T-cell membrane vesicles were prepared from the activated T cells to determine whether the vesicles with ICs expression could block their ligands and improve T cell function. Transmission electron microscopic results observed membrane vesicle-like bodies (Fig. 3A). Western blot results identified that the extracted T-cell membrane vesicles expressed T-cell membrane marker CD3 (Fig. 3B). Dynamic light scattering analysis revealed that the hydrodynamic diameter of T-cell membrane vesicles (M) and activated T-cell membrane vesicles (AM) were≈149.4 nm and 141.1 nm (Fig. 3C). Electrophoretic light scattering analysis showed that the zeta potential of M and AM were −31.7 mV and −31.5 mV **(**Fig. 3D). To better study the effect of AM on the T-cell cytotoxic activity under multiple immune checkpoint inhibition, high ICLs expressing 4T1-OVA/Luci tumor cells were prepared initially through IFN-γ stimulation, as detailed previously (Fig. S3, Supporting Information). Subsequently, these 4T1-OVA/Luci tumor cells were co-cultured with OT-I T cells for 24 h, followed by the evaluation of tumor viability. The results showed that the inclusion of AM notably augmented the cytotoxic activity of OT-1CD8^+^ T cells and secretion of functional cytokines (Fig. 3E and F and Fig S4A, Supporting Information). These results suggest that AM could potentially enhance T-cell cytotoxicity against cancer cells by impeding inhibitory signals from ICs.

The above results prompted us to design a nanocargo (AM-dLNP) based on the AM, which was expected to block multi-ICs inhibition pathways and serve as tumor-targeting carriers for STING agonist concurrently. The nanocargoes were prepared by coating diABZI-loaded nanoparticles of clinically approved liposomes with AM. The hydrodynamic diameter distribution of AM-dLNPs and control nanoparticles (M-dLNPs: non-actived T-cell membrane-decorated diABZI-loaded liposomes, AM-LNPs: AM-decorated empty liposomes) increased slightly, while the surface zeta potential decreased compared to that of the uncoated cationic liposomes (dLNPs: diABZI-loaded liposomes), in good agreement with data reported in the literature (Fig. 3G–J) [33]. The membrane proteins of ICs were also retained on AM-dLNPs after nanocargo synthesis (Fig. 3K). Further analysis by nanoscale flow cytometry revealed that multiple immune checkpoint proteins were localized to the nanoparticle surface. (Fig. 3L and M and Fig. S4B, Supporting Information). Transmission electron microscopic results further confirmed the particle size and distribution of AM on the surface of the diABZI-loaded liposomes (Fig. 3N). In addition, based on the results of high-performance liquid chromatography, the entrapment efficiency (EE%) of AM-dLNPs (58.05 ± 0.74 %) was slightly lower than that of control groups (dLNPs: 75.86 ± 1.34 %, M-dLNPs: 62.62 ± 1.01 %) (Fig. 3O and Fig. S4C and 4D, Supporting Information). Subsequent evaluation of diABZI release from AM-dLNPs under physiological conditions demonstrated a rapid release profile. Approximately 50 % of diABZI was released within 5 h across all groups, with near-complete release achieved by 24 h. And the dLNPs group exhibited a slightly faster release rate compared to the M-dLNPs and AM-dLNPs groups (Fig. S4E, Supporting Information). Given the rapid drug release profile associated with this liposome-based loading system, the nanoparticles were freshly prepared and used for all following studies. We further showed that there were no significant changes in the hydrodynamic diameter and zeta potential of AM-dLNPs over 5 days in serum, which was consistent with previous studies that cell membrane coating provided colloidal stability to nanoparticles in serum (Fig. S4F and 4G, Supporting Information) [34].

**Fig. 4:**
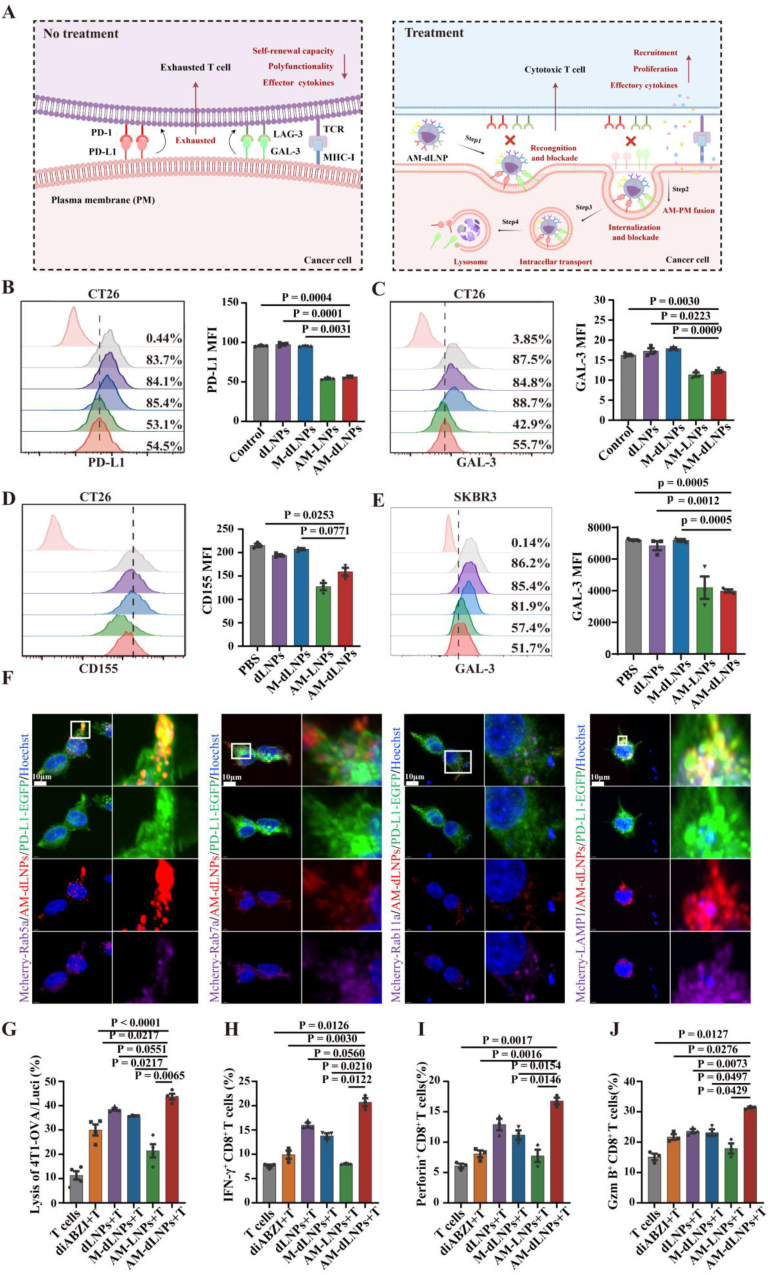
AM-dLNPs mediated multiple ICs blockade and enhanced T cell cytotoxicity. (A) Schematic illustration of blockade and internalization of PD-L1 and GAL-3 on cancer cell by AM-dLNPs. (B–D) Representative flow cytometric histograms (left) and bar graph (right) of the relative levels of membrane PD-L1 (B), GAL-3 (C), CD155 (D) on IFN-γ-pretreated (20 ng/ml, 24h) CT-26 cells after incubation for 4h with dLNPs, M-dLNPs, AM-LNPs and AM-dLNPs or PBS (control) (n = 3 independent experiments). (E) Representative flow cytometric histograms (left) and bar graph (right) of the relative levels of membrane GAL-3 on SKBR3 tumor cells after incubation for 4h with dLNPs, M-dLNPs, AM-LNPs and AM-dLNPs or PBS (control) (n = 3 independent experiments). (F) The dynamic colocalization of PD-L1 (green) with early endosomes (Rab5a, purple), late endosomes (Rab7, purple), circulating endosome (Rab11, purple) and the lysosomal marker Lamp1 in 293-T cells incubated with AM-dLNPs (red), as shown by confocal images, scale bar, 10 μm. The white box indicates the magnified region. (G) IFN-γ-pretreated (20 ng/ml, 24h) 4T1-OVA/Luci cells were treated with diABZI, dLNPs, M-dLNPs, AM-LNPs or AM-dLNPs for 4h (diABZI = 5 μg/ml), fowolled by co-culture with OT-1 T cells for 24 h at an effector: target ratio of 5:1 (n = 3 independent experiments). (H–J) Representative flow cytometric percentage of IFN-γ^+^ (H), Perforin^+^ (I) and Gzm B^+^ (J) in the aforementioned T cells, gated on CD3^+^ CD8^+^T cells (n = 3 independent experiments). All the data are presented as the mean ± S.D. The *p* values were determined by one-way ANOVA with Tukey's post-test for (B–E) and (G–J). (For interpretation of the references to color in this figure legend, the reader is referred to the Web version of this article.)

### 2.4 AM-dLNPs enhanced T cell cytotoxic activity

Given that antibody-mediated PD-1 blockade has been shown to induce internalization of PD-1 [35], we speculated that AM-dLNPs could be taken up by cancer cells along with multiple ICLs, leading to a substantial increase in T cell cytotoxic activity (Fig. 4A). By utilizing ICLs-expressed CT-26 cells, we further confirmed that the AM-derived vesicles triggered the internalization of membrane ICLs into cytoplasm, consequently reducing the expression of ICLs on the tumor cell membrane. Flow cytometric data revealed that AM-dLNPs and AM-LNPs treatment significantly reduced PD-L1 protein level with approximately 50 % decrease respectively, which was consistent with the results reported in the literature (Fig. 4B), which indicated that PD-1 expression level of the activated T-cell membrane vesicles was comparable to that of PD-1-transfected cell membrane in the literature [36]. Similarly, AM-dLNPs and AM-LNPs treatment also decreased the GAL-3 and CD155 (CD155: T cell immunoreceptor with Ig and ITIM domains (TIGIT) ligand) protein level by approximately 40 % and 30 %, respectively (Fig. 4C and D). Meanwhile, we prepared AM-decorated nanocargoes, in which AM was derived from human PBMCs. Consistently, flow cytometry demonstrated a significant reduction in detectable GAL-3 signals on human SKBR3 tumor cells and PD-L1 signals on human HT1080 tumor cells, which were treated with both AM-LNPs and AM-dLNPs compared to the control group, confirming effective ICLs blockade by human AM-derived vesicles (Fig. 4E and Fig. S5A, Supporting Information). Further confocal imaging revealed the intracellular colocalization of ICLs (PD-L1 or GAL-3) and AM-decorated nanocargoes in ICLs-expressed 4T-1 tumor cells, while a weak ICLs signal was observed in the cell cytoplasm treated with dLNPs and M-dLNPs (Fig. S5B and Fig. S5C, Supporting Information). We further evaluated the endocytosis efficiency of ICLs using the ratio between cytosolic and membranal ICLs fluorescence intensity. Treatment of 4T-1tumor cells with AM-dLNPs or AM-LNPs resulted in significant increase in the intral ICLs/m ICLs ratio compared to controls (Fig. S5D and Fig. S5E, Supporting Information). Subsequently, we monitored the dynamic trafficking of PD-L1 mediated by AM-derived nanovesicles in ICLs-expressed 293-T cells using confocal microscopy. To track the complex, cells were labelled with the early endosome marker Rab5a, late endosome marker Rab7, circulating endosome marker Rab11 and the lysosomal marker Lamp1. PD-L1 strongly colocalized with Rab5a, Rab7, Lamp1 but not with Rab11 (Fig. 4F), which suggested that AM-dLNPs-mediated PD-L1 blockade could lead to the degradation of PD-L1. Collectively, these results demonstrated that AM-guided nanocargoes could trigger the internalization of surface ICLs into cytoplasm and reduce ICLs expression on the tumor membrane.

**Fig. 5:**
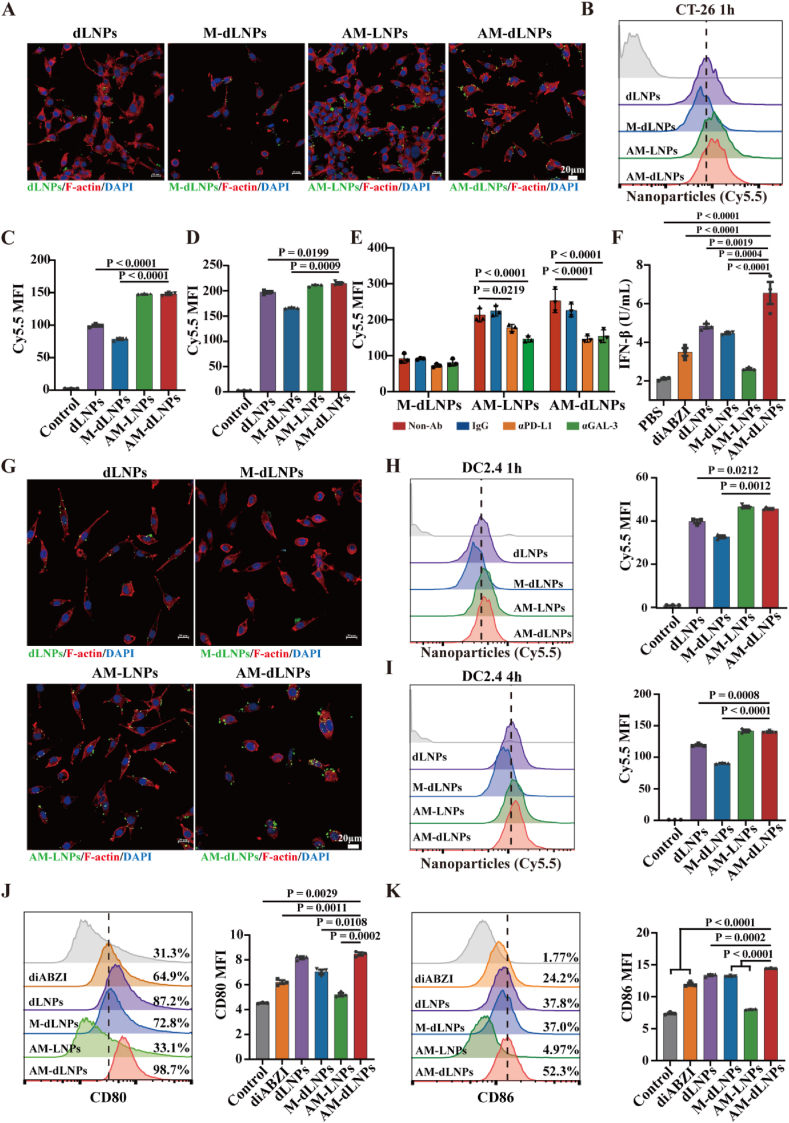
Cellular uptake of AM-dLNPs in vitro. (A) Representative confocal images of the intracellular localization of Cy5.5-labelled nanoparticles (green) incubated with IFN-γ-pretreated (20 ng/ml, 24 h) CT-26 cells. The cells were stained with DAPI (blue), and Phalloidin (red). Scale bars, 20 μm. (B, C) Representative flow cytometric histograms and bar graph of the cellular uptake of nanoparticles by IFN-γ-pretreated CT-26 cells after incubation with different nanoparticles for 1h. The nanoparticles were labelled with Cy5.5 (n = 3 independent experiments). **(**D**)** Representative flow cytometric bar graph of the cellular uptake of nanoparticles by IFN-γ-pretreated CT-26 cells after incubation with different nanoparticles for 4h. The nanoparticles were labelled with Cy5.5 (n = 3 independent experiments). (E) Flow cytometric analysis of cellular uptake of nanoparticles by IFN-γ-pretreated CT-26 cells blocked with anti-PD-L1 or anti-GAL-3 antibodies for 2 h prior to incubation with nanoparticles for 4 h. The nanoparticles were labelled with Cy5.5 (n = 3 independent experiments). (F) Levels of IFN-β in supernatants of CT-26 cells after treatment for 24 h with diABZI, dLNPs, M-dLNPs, AM-LNPs and AM-dLNPs or PBS. (n = 3 independent experiments). (G) Representative confocal images of the intracellular localization of Cy5.5-labelled nanoparticles (green) incubated with DC2.4. The cells were stained with DAPI (blue), and Phalloidin (red). Scale bars, 20 μm. (H) Representative flow cytometric histograms and bar graph of the cellular uptake of nanoparticles by DC2.4 after incubation with different nanoparticles for 1h. The nanoparticles were labelled with Cy5.5 (n = 3 independent experiments). (I) Representative flow cytometric histograms and bar graph of the cellular uptake of nanoparticles by DC2.4 after incubation with different nanoparticles for 4h. The nanoparticles were labelled with Cy5.5 (n = 3 independent experiments). The cells were stained with DAPI (blue), and Phalloidin (red). Scale bars, 20 μm. (J, K) Representative flow cytometric histograms (left) and bar graph (right) of DC maturation surface marker expression (CD80^+^, CD86^+^) in DC2.4 after 24 h treated with diABZI, dLNPs, M-dLNPs, AM-LNPs and AM-dLNPs or PBS (n = 3 independent experiments). All the data are presented as the mean ± S.D. The p values were determined by one-way ANOVA with Tukey's post-test for (C–D), (F) and (H–K); and by two-way ANOVA with Tukey's post-test for (E). (For interpretation of the references to color in this figure legend, the reader is referred to the Web version of this article.)

Next, we assessed the impact of AM-dLNPs on T-cell cytotoxic activity in the presence of immune checkpoint inhibition. 4T1-OVA/Luci cancer cells expressing ICLs were pre-treated with either AM-dLNPs or control nanoparticles for 4 h before co-culturing with OT-1 T cells for 24 h. The group treated with AM-dLNPs demonstrated the highest cytotoxic activity (Fig. 4G). This enhanced cytotoxicity in OT-1 T cells treated with AM-dLNPs was partly attributed to the immune checkpoint blockade mediated by the AM's ICs. Moreover, T cells treated with diABZI, AM-LNPs, M-dLNPs, or dLNPs exhibited increased cytotoxic activity compared to PBS-treated T cells (Fig. 4G and Fig. S6A, Supporting Information). The heightened cytotoxicity observed in these groups might be attributed to diABZI's known ability to enhance T cell activation by activating STING-mediated pathways and enhancing interferon-1 expression [37]. Specifically, diABZI-treated OT-1 T cells exhibited more than threefold higher cytotoxicity compared to OT-1 T cells. Consistent with the cytotoxicity results, various treatment groups showed elevated expression levels of effector cytokines compared to the PBS group. Notably, CD8^+^ T cells in the AM-dLNPs-treated group exhibited the highest expression levels of IFN-γ, Granzyme B (Gzm B), and Perforin (Fig. 4H–J and Fig. S6B–D, Supporting Information). Overall, AM-dLNPs promoted T-cell cytotoxic activation through AM-mediated immune checkpoint blockade and the activation of STING-dependent signaling pathways.

### 2.5 AM-dLNPs displayed outstanding drug-delivery characteristics

Cell membranes have emerged as promising drug-delivery vehicles for treating cancer and other diseases by utilizing membrane biomolecules, such as lipids and proteins [38]. Previous research has demonstrated that cell membranes can effectively target particular cells by binding ligands, facilitating efficient drug delivery [39]. Our screening process revealed that the AM exhibited high expression levels of multiple ICs and adhesion molecules. This characteristic suggests that the AM may have the ability to target multiple ligands on tumor cells, offering advantages over single-target drug delivery approaches. To make sure whether the AM-derived nanocargoes could play a role as we expected, it was necessary to evaluate the intracellular uptake behavior. Cy5.5 was used to label the nanoparticles by physical entrapment for visualization under flow cytometer analysis and confocal laser scanning microscopy. The nanocargoes were incubated with tumor cells for different time. After interaction with the cells, cellular uptake was detected to demonstrate the importance of the AM component for drug delivery. The observed images of CT-26 were illustrated in Fig. 5A, cells in the AM-dLNPs and AM-LNPs groups showed a stronger intracellular Cy5.5 fluorescent signals after 1 h of treatment. Further flow cytometric analysis confirmed a significant increase of Cy5.5 mean fluorescence intensity in the tumor cells after AM-dLNPs or AM-LNPs treatments compared to the control groups, indicating that nanoparticles obtained stronger transmembrane translocation ability after surface decoration with AM to become a complex (Fig. 5B–D, Fig. S7ASupporting Information). To determine whether the enhanced internalization of AM-decorated nanoparticles by tumor cells depended on ICs-ICLs binding, IFN-γ-pretreated CT-26 cells were subjected to antibody blockade against PD-L1 or GAL-3 prior to the assessment of nanoparticles uptake by flow cytometry. Compared to untreated groups, antibody blockade against PD-L1 or GAL-3 effectively attenuated the cellular uptake of the AM-decorated nanoparticles but not M-dLNPs, confirming the critical role of ICs-ICLs interactions in internalization process (Fig. 5E, Fig. S7B–D, Supporting Information). Studies have demonstrated that STING activation has been shown to induce tumor immunogenicity by activating the IRF3 (interferon regulatory factor 3)/IFN-β (interferon beta) pathway in tumor cells [40]. Thus, we next examined the IFN-β expression in tumor cells to judge the immunostimulatory potency of the several diABZI-loaded nanoparticles. The efficacy of diABZI-loaded nanoparticles in inducing IFN-β was detected using enzyme-linked immunosorbent assay. As expected, AM-dLNPs-treated tumor cells showed 3-fold higher IFN-β expression than PBS-treated cells and 2-fold higher IFN-β expression than free diABZI-treated cells, respectively (Fig. 5F).

The intense interaction between T and DC cells under physiological conditions relies on multiple receptor-ligand interactions [41], indicating that the AM may be well-suited for targeted drug delivery to DC cells. The DC2.4 images in Fig. 5G depicted enhanced intracellular Cy5.5 fluorescent signals in cells treated with AM-dLNPs and AM-LNPs groups after 4 h. Subsequent flow cytometric analysis validated a notable elevation in Cy5.5 mean fluorescence intensity in DC2.4 following treatment with AM-dLNPs or AM-LNPs compared to control groups (Fig. 5H and I). It is noteworthy that nanoparticles derived from AM demonstrated superior delivery efficacy compared to liposomes, a well-established and widely accepted carrier for drug delivery. Additionally, AM-derived nanoparticles exhibited enhanced biosafety profiles relative to liposomes, underscoring their promising clinical translational potential as drug carriers. Research indicated that STING agonists could exert the anti-tumor effect by promoting DC maturation, thereby further activating the immune system [42]. Consistently, AM-dLNPs induced significant up-regulation of CD80 and CD86 expression in DC2.4 compared with PBS and free diABZI groups (Fig. 5J and K). Thus, AM-dLNPs indeed performed better than dLNPs in enhancing the action of the loaded diABZI. Taken together, the AM-decorated nanoparticles possess remarkable transmembrane drug-delivery capability and further enhance the loaded-drug potency.

### 2.6 AM-dLNPs presented high tumor accumulation and reduced tumor growth

**Fig. 6:**
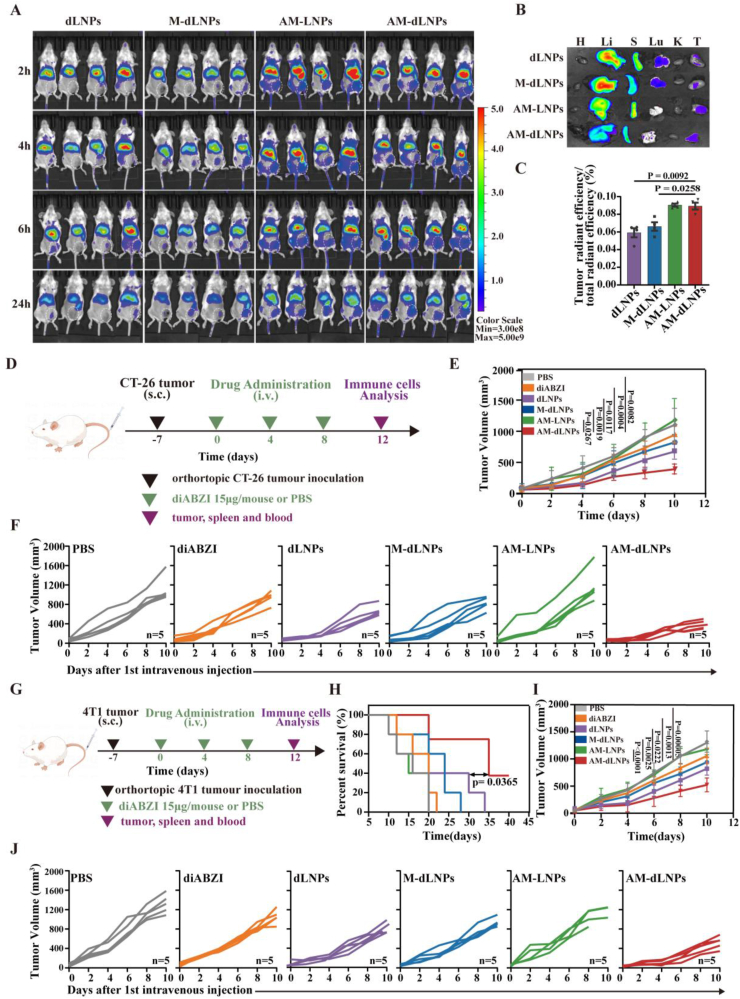
In vivo biodistribution and antitumor effects evaluation of AM-dLNPs. (A) In vivo fluorescence imaging of mice with CT-26 tumor receiving Cy5.5-labelled dLNPs, M-dLNPs, AM-LNPs, AM-dLNPs at 2 h, 4 h, 6h and 24h after administration (n = 4 mice). (B) Ex vivo fluorescence imaging of the major organs and tumors from the aforementioned mice euthanized 24 h postinjection (Cyanine dye Cy5.5: 0.1 mg per kg (body weight), intravenous injection, n = 1 mice). (C) Statistical analysis of the ration of fluorescence intensity at tumor tissues to total fluorescence intensity in vivo over time (n = 4 mice). (D) Experimental timeline and treatments for CT-26 tumor model (three intravenous injections, diABZI: 0.75 mg per kg (body weight)). (E, F) Average (E) and individual (F) CT-26 tumor growth curves of mice receiving different nanoparticles treatments (n = 5 mice). (G) Experimental timeline and treatments for 4T1 tumor model (three intravenous injections, diABZI: 0.75 mg per kg (body weight)). (H–J) Survival curves (H) and tumor growth profiles (I, J) of mice with 4T1 tumors receiving the indicated treatments (n = 5 mice). All the data are presented as the mean ± S.D. The *p* values were determined by two-way ANOVA with Tukey's post-test for (E) and (I); and by log rank (Mantel-Cox) test for (H).

ICs interact with ICLs, which are highly expressed in tumor cells, thereby playing a crucial role in tumor targeting. The presence of multiple ICs on the surfaces of AM-decorated nanoparticles may facilitate their more selective delivery to tumors, in contrast to single-target delivery methods. Consequently, we subsequently investigated the impact of the AM on the accumulation of the nanocargoes in the tumor in vivo. Cy5.5-labelled dLNPs, M-dLNPs, AM-LNPs, and AM-dLNPs were administered intravenously to mice bearing CT-26 tumors. Fluorescence imaging showed that the accumulation of Cy5.5-labelled AM-LNPs and AM-dLNPs in the tumor tissues progressively increased over a 24-h period following injection, in comparison to other treatment modalities (Fig. 6A). Simultaneously, we observed that fluorescence intensity peaked at approximately 6 h post-injection, followed by a gradual decline over 24 h. This was consistent with the expected pharmacokinetic profile: the nanoparticles reached peak systemic accumulation within a few hours, followed by a phase of clearance, leading to a gradual decrease in fluorescence intensity over time. Furthermore, the organs and tumors from the sacrificed mice were harvested 24 h after intravenous injection and taken for fluorescence measurement. As anticipated, AM-derived nanocargoes exhibited a greater degree of accumulation within tumor tissues among other groups (Fig. 6B), suggesting that AM-dLNPs might mitigate systemic toxicity through active targeting of tumor sites. The distribution of the nanocargoes within the liver and spleen of mice aligns with findings from previous research [43,44]. A quantitative analysis revealed that AM-decorated nanoparticles were significantly superior to dLNPs and M-dLNPs in the targeted delivery of diABZI to tumors. Furthermore, AM-dLNPs and AM-LNPs displayed comparable levels of accumulation in tumor (Fig. 6C). Therefore, despite the liver signal of the AM-dLNPs in the IVIS imaging was too strong for the tumor to be visualized, the targeting ability of AM-dLNPs was still confirmed. Tumor targeting by AM-dLNPs or AM-LNPs may involve two mechanisms. Firstly, nanoparticles are known to extravasate from the leaky tumor vasculature and preferentially accumulate within the tumor tissues [45]. Secondly, various ICs and adhesion-related proteins derived from the AM allow AM-dLNPs or AM-LNPs to target cancer cells in tumor tissues. According to previous studies, T cells are activated in the lymph nodes, and released by the lymph nodes, and then migrate to the tumor through adhesion and chemotaxis [46]. The above physiological process indicated that activated T cells should migrate into tumor sites more efficiently than non-activated T cells. Meanwhile, we also noted that multiple cell adhesion and migration genes such as Nectin2 and Alcam were up-regulated during T cell activation from the above RNA-sequencing results, which promoted T-cell adhesion and migration into tumor microenvironment. Furthermore, AM on the surface of nanocargoes can avoid the mononuclear-phagocyte-system-mediated clearance of the nanoparticles because of the inherent biological nature of cell membrane, thereby prolonging their circulation time and promoting tumor accumulation [47]. Thus, AM-dLNPs are expected to enhance therapeutic efficacy by improving tumor targeting while minimizing off-target adverse effects.

**Fig. 7:**
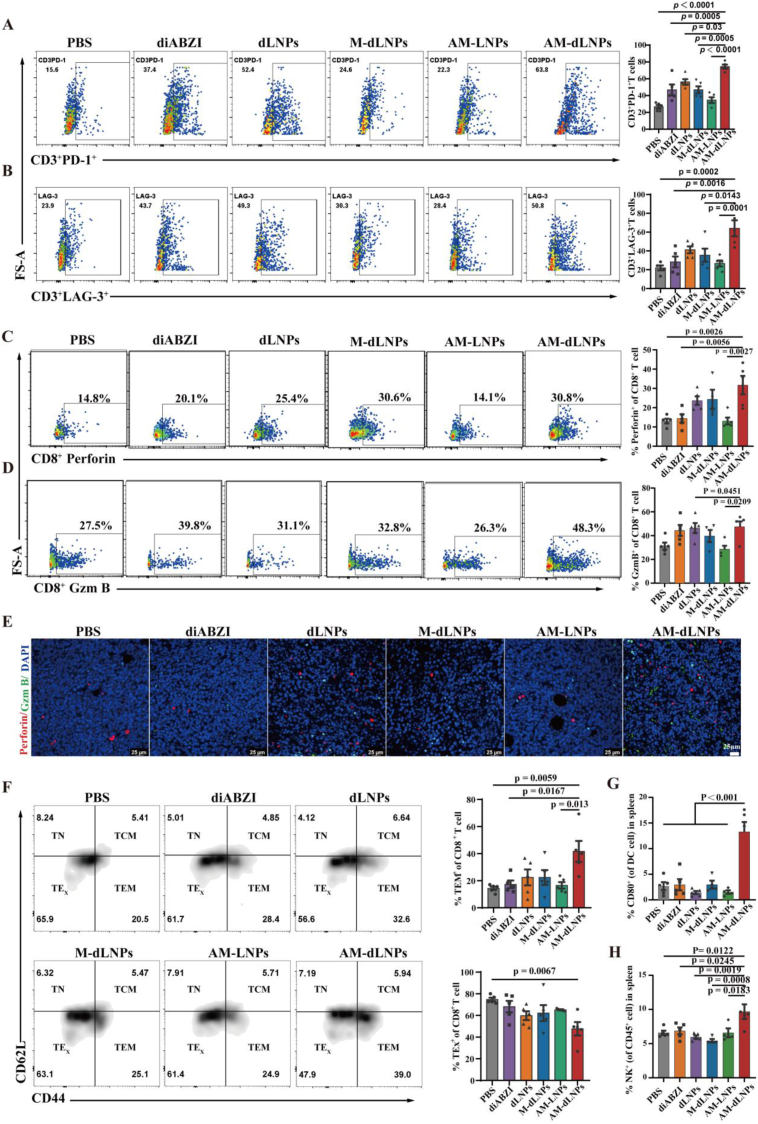
AM-dLNPs induced potent anti-tumor immune responses. (A–B) Representative flow cytometry plots (left) and statistical analysis (right) of T cell exhaustion markers PD-1 (A) and LAG-3 (B) on CD3^+^ T cells across different treatment groups by flow cytometry (n = 5 mice). (C–D) Representative flow cytometric plots and frequencies of Perforin (C) and Gzm B (D) that generated by tumor-infiltrating CD8^+^ T cells harvested on day 3 from bearing CT-26 mice after the last injection of the three-injection regimen. (n = 5 mice). (E) Representative IF images of tumor sections obtained from mice receiving three injections of the indicated treatments. Gzm B (green) and Perforin (red) were visualized using fluorescence-labelled antibodies. The nuclei were stained with DAPI (blue). Scale bars, 25 μm. (F) Representative flow cytometry plots and statistical analysis of naïve T cells (CD44^−^CD62L^+^), central memory T cells (CD44^+^CD62L^+^, TCM), effector memory T cells (CD44^+^CD62L^−^, TEM) and terminal exhausted T cells (TE_X_) within the TME across different treatment groups (n = 5 mice). (G, H) Representative flow cytometric frequencies of CD86^+^ DCs (G) and NK cells (H) in spleen harvested on day 3 from CT-26-bearing mice after the last injection of the three-injection regimen (n = 5 mice). All the data are expressed as mean ± S.D. The *p* values were determined by one-way ANOVA with Tukey's post-test for (A–D), (F) and (G, H). (For interpretation of the references to color in this figure legend, the reader is referred to the Web version of this article.)

Preclinical studies have demonstrated that pharmacological STING agonists can robust innate and adaptive immune responses against tumors, ultimately resulting in improved control of tumor growth [48]. Combining STING agonists with ICIs has the potential to enhance therapeutic outcomes by mitigating tumor-induced immunosuppression. Therefore, we assessed the AM-dLNPs platform untilizing a CT-26 murine colon cancer model to illustrate its potential for anti-tumor therapy. Upon achieving a tumor volume of 50–100 mm^3^, CT-26 tumor-bearing mice were intravenously administered with PBS, diABZI, dLNPs, M-dLNPs, AM-LNPs or AM-dLNPs on days 0, 4 and 8. (Fig. 6D). According to the cited literature and manufacturer, the effective dosage of diABZI utilized for anti-tumor therapy in murine models was 30 μg per mouse [49]. To circumvent the adverse toxicities and immune-suppressive effects associated with the systemic administration of diABZI, we halved the recommended dosage to 15 μg per mouse (0.75 mg/kg), because AM-dLNPs treated T cells produced a more robust killing and cytokines response than in free diABZI treated T cells prior in vitro experiments. The mice administered with AM-LNPs did not demonstrate any significant tumor growth inhibition when compared to the mice in the PBS group, whereas diABZI, dLNPs, and M-dLNPs treatment groups displayed partial suppression of tumors growth. Notably, the AM-dLNPs group exhibited the most pronounced tumor inhibition among all experimental groups (Fig. 6E and F). Unlike adoptive T cell therapy, the anti-tumoral effect of AM-dLNPs is characterized by tumor antigen-nonspecific responses. Considering this characteristic, we conducted an investigation to determine whether AM-dLNPs have anti-tumoral effects on a murine cold tumor (4T1), which is known for poor CTL infiltration (Fig. 6G). Consistent with the data of CT-26 tumor model, AM-dLNPs consistently inhibited tumor growth in the 4T1 breast cancer models as compared to the PBS-treated group (Fig. 6I and J). Additionally, therapy utilizing AM-dLNPs significantly prolonged the survival of mice (a 20-day increase compared to the PBS-treated group) (Fig. 6H), suggesting that diABZI delivery via the AM was effective in eliciting tumor antigen-nonspecific responses. The significantly enhanced tumor inhibition by AM-dLNPs, as opposed to dLNPs and M-dLNPs, indicates that combining multiple ICIs therapies with immunogenic inducers can effectively improve therapeutic efficacy. The superior therapeutic outcomes of AM-dLNPs over AM-LNPs may be attributed to their diverse therapeutic mechanisms beyond ICB, as well as the enhanced tumor-targeting capability of AM-dLNPs compared to AM-LNPs. These results collectively suggest that AM-dLNPs promote high tumor accumulation and potent anti-tumor immune responses.

### 2.7 AM-dLNPs triggered antitumor T cell immunity

Our in vitro studies indicated that AM-dLNPs reduced the surface expression of ICLs on tumor cells by triggering their internalization and subsequent lysosomal degradation. We subsequently evaluated the levels of intratumoral ICLs in mice bearing CT-26 tumors. The results indicated that the presence of diABZI resulted in the upregulation of ICLs (GAL-3, GAL-9 and PD-L1) in tumors compared to the PBS group (Fig. S8A, Supporting Information), consistent with the results reported in the literature. Fortunately, IHC results demonstrated that AM-dLNPs effectively suppressed the overexpression of ICLs on tumor cells compared with control groups (Fig. S8A, Supporting Information), which was consistent with the in vitro findings (Fig. S8A, Supporting Information). We further analyzed the ICs on T cells within the CT-26 tumors. Remarkably, both free diABZI and nanoparticles containing diABZI treatment significantly upregulated PD-1 andLAG-3 on CD3^+^T cells and CD8^+^T cells in tumor tissues (Fig. 7A and B and Fig. S8B–E, Supporting Information). The concomitant upregulation of immune checkpoints on T cells may underlie the limited efficacy of STING agonists by driving T cell dysfunction and tumor immune evasion. The results collectively supported the combination of a STING agonist and multi-target immune checkpoint blockades as a highly suitable anti-tumor approach. We then investigated if the downregulation of immune checkpoint ligands by AM could relieve immune checkpoint-mediated suppression and boost T cell cytotoxic function. To evaluate the function of T cells, the capacity of tumor-infiltrated T cells secreting Perforin and GzmB was detected by flow cytometry. In the tumors of the PBS group, Perforin^+^ (CD3^+^ CD8^+^ Perforin^+^) and GzmB^+^ (CD3^+^ CD8^+^ GzmB^+^) T cells accounted for only 14.8 ± 3.0 % and 27.5 ± 2.4 % of CD3^+^ CD8^+^ T cells (Fig. 7C and D). The proportion of Perforin^+^ and GzmB^+^ T cells hardly differed in AM-LNPs and PBS groups. With diABZI-loaded NPs treatment, the ability of T cells to secrete cytotoxic molecules was progressively increased. The largest percentage of Perforin^+^ T cells was observed in the AM-dLNPs group (30.8 ± 4.5 %), which was a 2.1-fold increase in tumors compared to PBS group (Fig. 7C). The percentage of CD8^+^ T cells capable of secreting GzmB was highest in AM-dLNPs group as well (48.3 ± 4.3 %), which was a 1.8-fold increase in tumors compared to PBS group (Fig. 7D). Similarly, the percentage of CD4^+^ T cells capable of secreting GzmB was highest in AM-dLNPs group as well (Fig. S8G, Supporting Information). Meanwhile, the immunofluorescence (IF) staining of tumor tissues, as expected, showed that AM-dLNPs upregulated the expression of Perforin and Gzm B in tumor tissues (Fig. 7E). In addition, a significant increase in effector memory T cells (TEM) was observed in the AM-dLNPs group, which was accompanied by a reduction in terminally exhausted T cells (TE_X_) (Fig. 7F). This TEM/TE_X_ shift suggests that AM-dLNPs may foster long-term antitumor immunity by favoring T-cell memory formation. The increased antitumor immunity of T cells observed in the AM-dLNPs group compared to that in dLNPs and M-dLNPs groups may be attributed to several factors. Previous research has demonstrated the capacity of AM-dLNPs to enhance the localization of diABZI at tumor sites, thereby facilitating T cell infiltration into tumors. Additionally, recent findings indicated that diABZI can enhance T cell cytotoxicity by activating the STING and TCR signaling pathways. Furthermore, AM-mediated multiple immune checkpoint blockades can further boost antitumor T cell immunity.

Given that previous studies have reported the role of diABZI in antigen presentation, DC maturation in vivo after the treatments was measured. The intratumoural percentage of mature DCs in the mice treated with AM-dLNPs was remarkably higher than that in PBS-treated mice (Fig. S8H, Supporting Information), and a similar trend was observed in the spleens (Fig. 7G and Fig. S8J, Supporting Information). STING-activating cyclic dinucleotides (CDN) enhanced NK cell activation, cytotoxicity, and antitumor effects as recently reported, independent of CD8^+^ T cells as recently research [50]. Consequently, we subsequently determined the proportion of NK cells in different treatment groups. Flow cytometry analysis revealed that AM-dLNPs resulted in enhanced infiltration of NK cells in the spleens (Fig. 7H, Fig. S8J, Supporting Information) and tumor tissues (Fig. S8I, Supporting Information) of mice bearing CT-26 tumors, indicating a favorable therapeutic outcome. Taken together, AM-dLNPs exhibited potent antitumoral efficacy by augmenting T cell-mediated cytotoxicity and promoting tumor immune cell infiltration.

### 2.8 AM-dLNPs showed excellent biosafety

**Fig. 8:**
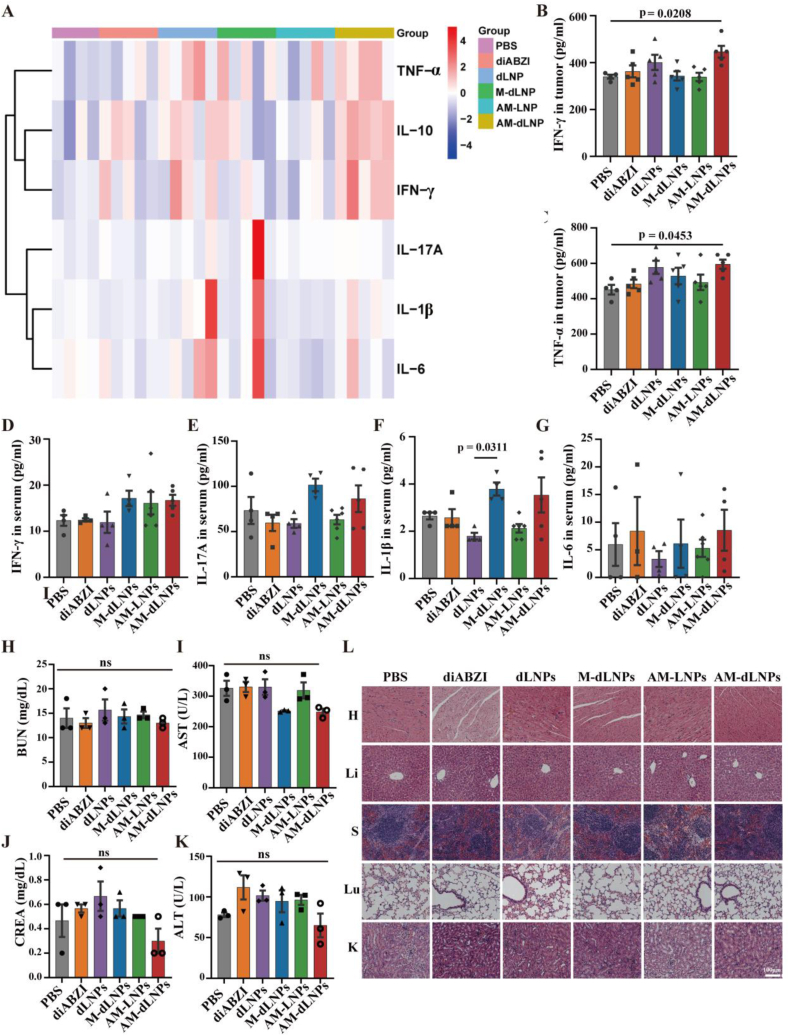
Biosafety profiles of AM-dLNPs in vivo. (A) Heatmap representation of indicated cytokines in tumor tissues harvested from CT-26-bearing mice on day 3 after the last injection of the three-injection regimen (n = 5 mice). (B, C) The quantification of intratumoural IFN-γ and TNF-α determined on day 3 after three injections of the indicated treatments (n = 5 mice). (D–G) The quantification of IFN-γ, IL-17, IL-1β and IL-6 in the serum collected from CT-26-bearing mice receiving the indicated treatments on day 3 after the third injection (n = 4 mice). (H–K) The concentrations of total protein CREA, BUN, ALT, AST in the serum collected from mice receiving the indicated treatments on day 3 after the third injection (n = 3 mice). (L) H&E staining images of the major organs (heart, liver, spleen, lung, and kidney) collected from mice receiving the indicated treatments on day 3 after the third injection. Scale bar: 100 μm. All the data are expressed as mean ± S.D. The *p* values were determined by one-way ANOVA with Tukey's post-test for (B–K).

The assessment of biosafety is a primary consideration in utilizing nanoparticle materials. A significant obstacle in the use of STING agonists for anti-tumor purposes lies in ensuring safety. Therefore, we conducted a comprehensive evaluation of the biosafety of AM-dLNPs. Excessive level of serum cytokines, termed cytokine storm, represents a significant adverse event commonly observed with the use of STING agonists in the context of cancer treatment. To confirm the cGAS-STING pathway activation and immune cell pro-inflammatory levels of the organism, the concentrations of a panel of cytokines in the tumor and serum were studied via the Bio-Plex ProTM assay. As illustrated in Fig. 8A–C andSupplementary Fig. S8A, Fig. S9A, the concentrations of IFN-γ, TNF-α, and IL-10 were significantly increased in the tumors of mice administered AM-dLNPs when compared to those treated with PBS. AM-dLNPs-induced cytokines secretion fostered a pro-inflammatory TME that facilitated T cell infiltration and antitumor function. Meanwhile, the concentrations of a panel of cytokines, including IFN-γ, TNF-α, interleukin-6 (IL-6), interleukin-17A (IL-17A), interleukin-1β (IL-1β) and interleukin-10 (IL-10) in serum were compared. No significant differences in the levels of IFN-γ, IL-17A, IL-1β, and IL-6 were observed among the groups at 3 days post-treatment, in which the IL-1β and IL-6 are the key mediators of cytokine release syndrome induced damage (Fig. 8D–G) [51]. And AM-dLNPs-treated mice showed an elevated TNF-α and IL-10 level in serum, which regulated the body's inflammatory response (Fig. S8B and S8CFig. S9B and S9C, Supporting Information). Meanwhile, serum cytokine levels were measured on day 1 after the third dose to monitor for potential acute cytokine spikes. Results showed that treatment with free diABZI resulted in a significant increase in the levels of TNF-α, IL-10, and IL-1β compared to the experimental group. No significant differences were observed between the AM-dLNPs and PBS groups (Fig. S8D–S8IFig. S9D–S9I, Supporting Information). The results suggested that AM-dLNPs primarily induced localized inflammation within the tumors, from which the serum cytokines are probably originated.

Subsequently, we examined other biological indicators related to biosafety. The body weight of the tumor-bearing mice did not exhibit any significant changes following the treatments, indicating that AM-dLNPs were well tolerated by the mice under the current regimen (Fig. S8GFig. S9G, Supporting Information). The biochemical analysis of blood indicators in mice subjected to different treatments, including blood creatinine (CREA), blood urea nitrogen (BUN), alanine aminotransferase (ALT) and aspartate aminotransferase (AST) showed no significant differences, indicating that the side effects associated with administered nanoparticles were negligible at the specified dosage (Fig. 8H–K). Furthermore, we performed histopathological analysis utilizing hematoxylin and eosin (H&E) staining. Experimental data revealed that cardiotoxicity was exclusively observed in the diABZI treatment cohort, possibly because free small-molecule diABZI is more likely to accumulate in the heart than its nanoparticle-encapsulated counterparts (Fig. 8L). To further evaluate the potential cardiotoxicity of the STING agonist-based regimens, cardiac tissues were subjected to TUNEL staining. The results demonstrated that, compared to PBS, none of the treatment groups induced significant cardiotoxic effects (Fig. S8KFig. S9K, Supporting Information). Taken together, these results demonstrated that the systemic administration of AM-dLNPs can safely and effectively deliver immune-stimulatory STING agonists to the tumor microenvironment.

The data collectively demonstrated that treatment with AM-dLNPs induced robust immune responses in diverse tumors by inhibiting immune checkpoints and eliciting potent cytotoxic T lymphocyte responses, resulting in optimal tumor suppression. Therefore, we propose that AM-dLNPs represent an effective nanotherapeutic approach for overcoming immunosuppression and enhancing immune activation. This biomimetic nanosystem utilizes endogenous cell membranes as a natural platform for various biological processes and functional protein expression. AM-dLNPs exhibit a promising spatiotemporal efficacy, capable of effectively targeting and treating tumors.

Cell membrane has emerged as a highly promising platform for drug delivery and various biological applications. Accumulating evidence indicates that the molecular composition and functional characteristics of the cell membrane can vary significantly across different cellular states. For instance, multiple studies have reported that membrane vesicles derived from apoptotic or irradiated tumor cells displayed elevated antigen expression and enhanced immunogenicity [52,53]. It is well established that primary T cells exhibit distinct expression profiles and functional behaviors depending on their activation status. Under certain activated conditions, T cells upregulate multiple ICs to maintain immune tolerance. In this study, we initially screened activated T cells characterized by elevated ICs expression corresponding to specific T cell states. Subsequently, we isolated activated T-cell membrane vesicles from these cells, which were demonstrated to effectively block multiple ICs, thereby enhancing the antitumor efficacy of T cells. Building on these findings, we developed a novel antitumor strategy integrating a STING agonist into a membrane-based platform. This approach elicited marked antitumor efficacy. However, when investigating the tumor-targeting capability of the resulting nanomedicine, we observed only a moderate level of tumor-targeting efficacy. We hypothesize that this may be attributable to the inversion of the inner membrane during vesicle preparation, leading to a reduced surface density of immune checkpoint molecules—a notion supported by our nanoflow cytometry data. Additionally, due to the complexity of protein composition on the cell membrane surface, the potential contributions of other membrane proteins remain to be elucidated. Nevertheless, the collective evidence from our study validated the overall antitumor benefit of the AM-based platform. While this foundational work establishes its promise, the clinical translational potential of the AM-based platform remains to be fully elucidated. Future studies should prioritize a direct comparison with established ICI therapies to rigorously assess its relative therapeutic value.

## 3 Conclusion

Here, we developed an activated T-Cell membrane-based multifunctional immunotherapy platform that was shown to effectively block multiple ICs and serve as a tumor-targeting carrier for immunogenic inducers. This unique membrane endows nanoparticles with the capability of overcoming STING agonist-regulated multigenic resistance to ICIs by specifically recognizing and blocking multiple ICLs. Most notably, the optimal anti-tumor therapeutic benefit was achieved with half of the recommended diABZI dose in the literature, reducing the risk of potential side effects. Regarding the future clinical translation of the AM-derived platform, it is critical to consider good manufacturing practice upgrade and standardization issues. In terms of the source materials, adoptive cell transfer (a well-established clinical treatment method) guarantees the supply of ICs-expressing T cell membrane vesicles of clinical quality and quantity. The platform also benefits from existing infrastructures and logistics for nanotherapeutics especially drug-bound liposomes [54]. These technological advances in nanotherapeutics and personalized medicine manufacture offer encouraging prospects for the clinical transformation of AM-guided nanocargoes. The versatility of the AM-platform also enables it to be applied to targeted delivery of other immune-stimulating agents for synergistic anti-tumor immunotherapies.

## 4 Experimental section

### 4.1 Materials

diABZI (cat.no. HY-112921A) and Cy 5.5 dye (cat.no. HY-D0924) were purchased from Med Chem Express. SM102 was obtained from HuBei Innerse Biotechnology. DMG-PEG2000 and DODAP were obtained from ShangHai Ponsure Biological. Cholesterol, Ammonium sulfate and ethanol were purchased from Macklin. Trypsin-EDTA (cat.no.25200072, 0.25 %), RPMI 1640, Dulbecco's modified Eagle medium (DMEM), Penicillin-Streptomycin (cat.no. 15140-122, 100 × ), Pierce Protease and Phosphatase Inhibitor Mini Tablets (cat.no. A32961, EDTA-Free), HEPES (cat.no. 15630130, 1M), GlutaMax ™ (cat.no. 35050061, 100 × ), MEM Non-Essential Amino Acid (MEM NEAA, cat.no. 11140050, 100 × ) and Sodium pyruvate (cat.no.11360070, 100 mM) were obtained from ThermoFisher. Fetal bovine serum (FBS, FSD590) was purchased from ExCell. β-Mercaptoethanol (cat.no. 60-24-2, 55 mM) was purchased from RHAWN. Recombinant Murine IL-2 (cat.no.212-12), Recombinant Murine IL-4 (cat.no.214-14),Recombinant Murine GM-CSF (cat.no.315-03) and Recombinant Murine IFN-γ (cat.no.315-05) were obtained from PEPROTECH.TSA Fluorescence Double Staining Kit were obtained from Abmart (cat.no. MFIHC03B). EasySep™ Mouse T Cell Isolation Kit was purchased from STEMCELL Technologies (cat.no. 19851A). QuantiCyto^R^ Mouse IFN-β ELISA Kit was obtained from NeoBioscience. DAB Horseradish Peroxidase Color Development Kit (cat.no. ZLI-9017), Phosphate-buffered saline (PBS, cat.no.ZLI-9061, pH7.3) and Citric Acid Buffer (cat.no.ZLI-9064, pH6.0) were purchased from ZSGB-BIO. The Antifade Mounting Medium with DAPI (cat.no. P0131) and Calcein AM Cell Viability Assay Kit (CCK-F, cat.no. C2013FT) was purchased from Beyotime. Isoflurane was purchased from Orbiepharm.

### 4.2 Mice

BALB/c mice and OT-1 mice (male and female, six to eight weeks) were purchased from the Laboratory Animal Services Center of Chongqing Medical University. All mice were housed under specific-pathogen free conditions with normal mouse chow, a 12 h light-dark cycle and controlled temperature (20–22 °C) and humidity (40–60 %). All animal studies were handled according to protocols approved by the Ethics Committee of Chongqing Medical University (Approval No. IACUC-CQMU-2024-0567).

### 4.3 Cell lines and cells

SKBR3 and HT1080 cell lines were obtained from ATCC and cultured in Dulbecco's modified Eagle medium supplemented with 10 % fetal bovine serum (FBS). The luciferase-labelled murine BC cell line 4T1-OVA/Luci,4T1, murine colorectal carcinoma cell line CT-26, murine dendritic cell line DC2.4 were purchased from Shanghai Cell Bank of Chinese Academy of Sciences and cultured according to the supplier's recommendations, supplemented with 10 % FBS and 1 % Penicillin-Streptomycin. All the cells were cultured at a 37 °C incubator under 5 % CO_2_ and 90 % humidity.

### 4.4 Antibodies

The fluorescence-labelled antibodies for flow cytometry (FCM) were against mouse CD45 (Biolegend: cat. no. 103138), mouse CD3 (Biolegend: cat. no. 100203), mouse CD4 (Biolegend: cat. no. 100433), mouse CD8 (Biolegend: cat. no. 100729), mouse intracellular perforin (Biolegend:cat.no. 154405), mouse intracellular granzyme B (Biolegend:cat.no. 372204), mouse intracellular IFN-γ (Biolegend:cat.no. 505825),mouse PD-1 (Biolegend:cat.no. 135217), mouse TIM-3 (Biolegend:cat.no. 134003),mouse LAG-3 (Biolegend:cat.no. 125209),mouse TIGIT (Biolegend:cat.no. 142107),mouseF4/80 (Biolegend:cat.no.123111),mouse CD11c (Biolegend:cat.no. 117329), mouse CD80 (Biolegend:cat.no.104713), mouse CD86 (Biolegend:cat.no. 105014), mouse MHC-II (Biolegend:cat.no.116507), mouse CD49 (Biolegend:cat.no. 108907), mouse PD-L1 (Biolegend:cat.no.124307), human/mouse Galectin-3 (Biolegend:cat.no. 125405), mouse Galectin-9 (Biolegend:cat.no. 136103), mouse CD155 (Biolegend:cat.no.131507). mouse CD44 (Biolegend:cat.no. 103027), mouse CD62L (Biolegend:cat.no. 161269). human CD274 (Biolegend:cat.no. 329705). STING (D2P2F) Rabbit Monoclonal Antibody (Cell signaling Technology: cat.no. 13647S), PD-1/CD279 Monoclonal antibody (Proteintech: cat.no. 66220-1-Ig), LAG-3 Polyclonal antibody (Proteintech:16616-1-AP), and Recombinant Anti-TIM-3 antibody (Abcam: EPR22241), PD-L1 Rabbit Polyclonal Antibody (Abiowell: cat.no. AWA59204), Galectin-3 Rabbit Polyclonal antibody (Abiowell: cat.no. AWA10115) and Galectin-9 Polyclonal antibody (Proteintech: cat.no.17938-1-AP) were used for IHC. Perforin polyclonal antibody (Proteintech: cat. no. 13588-1-AP, 1:50), Granzyme B polyclonal antibody (Proteintech: cat. no. 14580-1-AP, 1:500) and secondary antibody SignalEnhance Goat anti Rabbit (Abmart: cat.no. A10029, Poly HRP) were used for Immunofluorescence Analysis. CD3 Monoclonal Antibody (ThermoFisher: cat.no.16-0032-82) and CD28 Monoclonal Antibody (ThermoFisher: cat.no.16-0081-82) were used for T cells activation and exhaustion.

### 4.5 STING and immune checkpoint expression in lung cancer patients

To identify the association between intratumoural STING signature and immune checkpoint, lung tumor specimens from 20 patients at the First Affiliated Hospital of Chongqing Medical University were collected for Immunohistochemistry (IHC), as approved by the hospital's ethics committee (Approval No. 2024-076-01).

### 4.6 Effect of diABZI on tumor infiltration of immune cells and its therapeutic efficacy

CT-26 tumor model: Subcutaneously inoculated CT-26 cells (1 × 10^6^ cells/mouse) in BALB/c mice (16 ± 2g, 6–8 weeks) (n = 5). When it was about 100 mm^3^, the tumor-bearing mice were treated with diABZI (0.75 mg/kg (body weight) (15 μg per injection), three intravenous injections) or PBS on day0, 4, 8. Tumor volume was calculated according to Eq: Tumor volume = length × width × width/2. The tumors were collected three days after the last injection, weighed, cut into small blocks and gently crushed against a nylon screen. The collected cells were stained with the corresponding antibodies to determine the amounts of CD45^+^ immune cells, CD3^+^ T lymphocytes, NK cells, DCs, macrophages and the expression of granzyme B, perforin, PD-1, LAG-3, TIM-3 in the tumors via flow cytometry (FCM) using Flowjo_V10 software.

### 4.7 Mouse T cells isolation, activation and screening

Peripheral blood or spleens from BALB/c mice or OT-1 mice (6–8 weeks old) were collected, and single cell suspension was prepared in mouse Lymphocyte Separation Medium. Primary mouse T cells were enriched with EasySep™ Mouse T Cell Isolation Kit. For activated T cells, 24-well Corning-Costar plates were coated with 1 μg/mL anti-CD3 monoclonal antibodies in PBS for 16–24 h at 4 °C before seeding T cells. Immediately after magnetic isolation, T cells were resuspended in complete medium (RPMI1640 medium supplemented with 10 % FBS, 25 mM HEPES, 1 × GlutaMax, 100 IU/mL penicillin, 100 μg/mL streptomycin, 55 μM β-Mercaptoethanol, 1 × MEM NEAA, 1 mM Sodium pyruvate, 200U/ml IL-2),and seeded in the antibody-coated plate at 1.5 × 10^6^-2 × 10^6^ cells/well together with soluble 1 μg/mL anti-CD28 monoclonal antibodies. Cells were kept on these activation plates for 48 h at the beginning of all experiments. Subsequently, activated T cells were passaged onto a new plate every two days and maintained in 200U/ml IL-2 alone. On day 6, the cells were rest 24 h in IL-2-free complete medium. After that, cells were passaged onto a fresh anti-CD3-coated plate and activated 48 h in complete medium with 1 μg/mL anti-CD28, passaged onto a new plate for another 24 h in complete medium. The cells were analyzed every day via FCM, as described in the Results.

### 4.8 RNA-seq

We performed RNA-seq analysis using primary T cells in good growth status under different culture conditions. T cells were activated and expanded for 6 days in tissue culture-treated 24-well plates (2 × 10^6^/well) with anti-CD3 mAb and anti-CD28 mAb in T cell medium. Next, the T cells were rested for 24h and then reactivated for 3 days. Toal RNA was prepared from activated T cells using Trizol. Primary T cells harvested from the spleen by magnetic cell sorting were served as controls. Each experimental group had three biological replicate samples for RNA-seq experiments. The subsequent sequencing library construction and QuantSeq3'mRNA-seq experimental procedures were performed at Beijing Novogene Co., Ltd. The GO functional enrichment analysis of differentially expressed genes was evaluated by Fisher's exact test, where P ≤ 0.05 indicates a statistically significant level of enrichment analysis. For the enrichment analysis, we focused on the cell membrane-related gene analysis according to the website (https://membranome.org/).

### 4.9 Isolation of T-cell membrane

The plasma membrane was collected from T cells according to previously reported methods but with modifications. Briefly, specific activated T cells were collected grown in 24-well plates, harvested, and washed three times with PBS (pH7.4). The pellet was suspended in hypotonic lysis buffer containing 20 mM Tris-HCl (pH7.5), 10 mM KCl, 2 mM MgCl_2_, and Pierce Protease and Phosphatase Inhibitor Mini Tablets (EDTA-Free, 1 tablet per 10 mL of solution) in an ice bath for 3h, and disrupted by sonication 5min at a power of 80W (sonication for 3s, intervals for 5s on ice). The homogenate was then centrifuged at 7000g and 4 °C for 10min to remove intact cells and mitochondria. The supernatant was further centrifuged at 148000g and 4 °C for 45min. The un-activated T cells membrane was isolated using the same method. After centrifugation, the membrane was collected as a pellet, suspended in 0.2 mM EDTA and stored at −80 °C for subsequent experiments. Protein content in the isolated membrane was determined using Bradford regent according to the manufacturer's protocol. The protein content in purified membrane from 1 × 10^8^ cells was 0.3 mg.

### 4.10 Preparation of positively charged liposomes loaded with diABZI

The precursor solution for the synthesis of stabilized micromeric liposomes was initially formulated, incorporating an ammonium sulfate solution as the aqueous phase and a lipid molecule alcohol solution as the lipid phase. The aqueous phase consisted of a 25 mmol/L ammonium sulfate solution, while the lipid phase was composed of an ethanol solution containing lipid components such as SM102, cholesterol, DMG-PEG2000, and DODAP, in a molar ratio of 6:6:2:1. The lipid mixture was prepared by combining the following components per mL of total volume: 286 μL of SM-102 (20 mg/mL), 154 μL of cholesterol (20 mg/mL), 86 μL of DODAP (20 mg/mL), 167 μL of DMG-PEG2000 (20 mg/mL) and 307 μL ethanol. The mixture was vortexed thoroughly until a clear solution was obtained, yielding a final total lipid concentration of 10 mg. The precursor solution was introduced through distinct inlets of a microfluidic chip, with the aqueous-phase solution and lipid-phase solution being injected from their respective ports, and a collection device positioned at the outlet. The mixing of these solutions occurred within the mixing channel of the microfluidic chip. A micro syringe pump system (Chemyx micro syringe pump, USA) was employed to facilitate the mixing of the raw material solutions, with the total flow rate adjusted to establish the desired flow rate ratio between the ammonium sulfate solution and the lipid molecule ethanol solution. The flow rates for both the aqueous and lipid phases were maintained between 0.1 mL/min and 2 mL/min. The resultant products were collected at the outlet of the main microchannel of the microfluidic chip, and the final liquid products were isolated using ultrafiltration centrifuge tubes. Centrifugation was conducted at 4000 r/min for a duration of 20 min, yielding the prepared blank cationic liposome (LNP).

### 4.11 Preparation of AM-dLNPs and characterization

AM-dLNPs were fabricated by coating dLNPs with specific activated T cells membrane (AM) by a direct extrusion method. Briefly, the purified AM from 1 × 10^8^ cells was mixed with 1.0 mL of dLNPs with diABZI concentration of 0.9 mg/mL, and sequentially extruded through polycarbonate membrane with the pore size of 400 nm and 200 nm to prepare AM-dLNPs. M-dLNPs was prepared using the same method with non-activated T cells membrane (M). The size distribution and zeta potential of nanoparticles were determined by dynamic light scattering measurement (Malvern ZEN 3600 Zeta sizer, UK). The mean diameter and zeta potential of nanoparticles in 50 % FBS were detected from day 1 to day 5 to evaluate their stability over time. Meanwhile, the morphology was imaged under transmission electron microscope (Thermo Fisher Scientific CDLtd, Talos F200S) after negative staining with phosphotungstic acid solution (1 %, w/v). The encapsulation efficiency (EE%) was measured using high-performance liquid chromatography (SHIMADZU Corporation, LC-20A). The encapsulation efficiency was calculated using the following formulas: EE% = Weight of diABZI losded in liposome/Total weight of diABZI used initially × 100 %. Moreover, the cell membrane surface markers (CD3, PD-1, TIM-3, LAG-3) were characterized by Western blot.

### 4.12 Release kinetics of AM-dLNPs

The nanoparticle sample was placed in a 3500 Da molecular weight cutoff dialysis bag and immersed in 200 mL of 0.1 M PBS (pH 7.4) containing 0.5 % Tween-80. The release medium was maintained at 37 °C with constant magnetic stirring at 300 rpm. At predetermined time intervals (10 min, 30 min, 1 h, 2 h, 4 h, 6 h, 8 h, 16 h, 24 h, 36 h, and 48 h), 200 μL aliquots were collected and immediately replaced with an equal volume of fresh medium to maintain sink conditions. The collected samples were diluted with methanol and centrifuged at 14,000×*g* for 10 min. The supernatant was then analyzed by HPLC to determine the drug concentration.

### 4.13 Multiple ICs blocking and internalizing in vitro

To verify the level of PD-L1, Gal-3, GAL-9, CD155 increased by IFN-γ, CT-26 and 4T1 cells were cultured 24h with the presence or absence of 20 ng/mL IFN-γ. Then the expression of immune checkpoint of the two groups was tested using FCM (Beckman). For blocking experiments, IFN-γ-pretreated CT-26 cells were treated with AM-dLNPs, AM-LNPs,M-dLNPs, dLNPs or PBS for 4h in 24-well Corning-Costar plates. The cells were collected, washed with PBS and stained with the corresponding antibodies before FCM analysis. For internalizing experiments, The IFN-γ-pretreated 4T1 cells (8 × 10^4^) were seeded in a pretreated 30 mm Nunc glass-bottom dish and allowed to attach overnight. The medium was replaced with fresh medium containing Cy5-labelled AM-dLNPs, AM-LNPs,M-dLNPs and dLNPs (Cy5: 4ug/ml). After incubation for 4 h, PE PD-L1 antibody and PE GAL-3 antibody were added to stain the membrane PD-L1 and GAL-3, respectively. Subsequently, the cells were fixed and permeabilized, and PE/Cyanine7 PD-L1 antibody, Alexa Flour 488 GAL-3 antibody and DAPI were added to stain theintracellular PD-L1, GAL-3 and nucleusrespectively. The cells were then washed and imaged using confocal laser scanning microscopy (CLSM, ZEISS, LSM 900).

### 4.14 Tumor cell killing induced by autologous T cells in vitro

OT-1 T cells were activated 72h as described above. FN-γ-pretreated (20 ng/ml, 24h) 4T1-OVA/Luci cells were treated with diABZI, dLNPs, M-dLNPs, AM-LNPs or AM-dLNPs for 4h (diABZI: 5 μg/ml), fowolled by co-culture with OT-1 T cells for 24 h at an effector: target ratio of 5:1(n = 3). The viability of 4T1-OVA/Luci cells was detected by Calcein⁃AM staining or the luciferase reporter assay system. The ability of AM-dLNPs to enhance T cells cytotoxicity was demonstrated by detecting the levels of IFN-γ, Granzyme B, Perforin via FCM.

### 4.15 Cellular uptake in vitro

We compared the uptake efficiency of AM-dLNPs, AM-LNPs, M-dLNPs and dLNPs. We seeded DC2.4 and IFN-γ-pretreated CT-26 cells into confocal culture dishes and 24 -well plates at 8 × 10^4^ cells every well and allowed to attach overnight. The medium was replaced with fresh medium containing Cy5.5-labelled AM-dLNPs, AM-LNPs,M-dLNPs and dLNPs (Cy5.5: 4ug/ml). After incubation for 1h and 4 h, the cells with different treatments were observed via CLSM and FCM.

### 4.16 Cytokine detection

The level of IFN-β in the cell medium was measured using ELISA kits according to the manufacturer's protocols. CT-26 cells (2 × 10^5^ cells in 500ul DMEM; n = 3) were seeded in 96-well plates and allowed to attach overnight. The medium was replaced with fresh medium containing AM-dLNPs, AM-LNPs,M-dLNPs, dLNPs and diABZI (5 μg/ml) or PBS. After incubation for 24 h, the medium was collected for analysis.

### 4.17 The activation of DCs in vitro

DC2.4 cells (2 × 10^5^) were seeded in 24-well plates and allowed to attach overnight. The medium was replaced with fresh medium containing AM-dLNPs, AM-LNPs,M-dLNPs, dLNPs and diABZI (5 μg/ml) or PBS. Following incubation for 24h, cells were scraped, washed with cold PBS, and stained with markers of DC activation (CD80, CD86) followed by FCM.

### 4.18 Accumulation of nanoparticles in tumor tissues

A total of 1 × 10^6^ CT-26 cells were introduced by subcutaneous injection into the right flanks of Balb/c mice. Upon reaching a tumor volume of 50–100 mm^3^, the tumor-bearing mice were randomly assigned to different four groups and administered intravenously Cy5.5-lablled AM-dLNPs, AM-LNPs, M-dLNPs and dLNPs (n = 4, Cy5.5: 0.1 mg per kg (body weight)). Fluorescence signals in mice and in the tumors were monitored at 2, 4, 6, and 24 h post-injection with an IVIS Spectrum Imaging System (PerkinElmer, USA). After in vivo imaging, one mouse in each group were sacrificed, and the tumors together with other major organs (heart, liver, spleen, lungs, kidneys) were collected for ex vivo imaging. The fluorescence signals from different organs were analyzed with IVIS software.

### 4.19 Antitumor therapy in mice bearing CT-26 tumor

To investigate the anti-tumor effect of AM-dLNPs, the mice bearing CT-26 tumors (tumor volume of 50–100 mm^3^) were intravenously injected (i.v.) with 100 μL nanoparticles or free diABZI (0.75 mg diABZI/kg) on day 0, 4, and 8 using PBS as a control (n = 5). Tumor volume and body weight of the mice were recorded every two days. Mice were designated dead when the mice died, body weight loss was over 15 %, or tumor volume was over 2000 mm^3^. The tumors and spleen were collected on day 3 after the last injection, weighed, cut into small blocks and gently crushed against a nylon screen. The collected cells were stained with the corresponding antibodies to determine the amounts of CD45^+^ immune cells, CD3^+^ T lymphocytes, CD4^+^ T cells, CD8^+^ T cells, NK cells, DCs in the tumors and spleen via FCM. The serum samples and tumors were collected for further experiments three days after the last injection.

### 4.20 In vivo safety evaluation

For the biosafety evaluation, 1 × 10^6^ 4T1 cells were introduced into the right flanks of Balb/c mice by subcutaneous injection. Upon reaching a tumor volume of 50–100 mm^3^, 4T1 tumor-bearing mice were intravenously injected with 100 μL nanoparticles or free diABZI (diABZI: 0.75 mg/kg) on day0, 4, and 8 using PBS as a control (n = 10) through the tail vein. Tumor volume, body weight and rate of survival of the mice were recorded every two days.

### 4.21 Measurement of cytokines and serum chemistry parameters

Tumor suspension and serum samples from CT-26 tumor-bearing mice receiving three intravenous injections of different treatments (diABZI: 0.75 mg/kg) were collected on day 3 after the last treatment. The snap-freeze process was adopted using liquid nitrogen and the samples were stored at −80 °C until further analysis. The cytokine levels were determined via Bio-Plex Pro™ Assays according to the manufacturer's protocol. Serum samples were also sent to the First Affiliated Hospital of Chongqing Medical University for analysis of serum chemistry parameters.

### 4.22 Immunofluorescence analysis

Immunofluorescence evaluations were performed to examine Granzyme B, Perforin and PD-L1 in tumor tissue using TSA Fluorescence Double Staining Kit. Briefly, the harvested tumors were initially placed in 4 % paraformaldehyde for fixation. They were then dehydrated, embedded, and sliced. The sections were deparaffinized and immersed in 3 % methanol hydrogen peroxide for 10 min at 25 °C. Following three washes with PBS buffer, the slices were submerged in citric acid buffer (0.01 M, pH 6.0) and microwave-heated to boiling for antigen retrieval. After three additional washes with PBS, the slices were blocked by incubating them with goat serum for 30 min at 25 °C. A Perforin antibody (1:500), a Granzyme B antibody (1:50) and a PD-L1 antibody (1:50) were diluted with 5 % goat serum and added to the sections overnight at 4 °C, followed by incubation with a secondary antibody (goat anti-rabbit, poly HRP) at 25 °C for 50 min, then the Tyramide signal amplification solution was added to the sections, the nuclei were stained by DAPI, and imaged by a Confocal laser scanning microscope.

### 4.23 IHC study and histological analysis

Immunostaining was completed on paraffin-embedded tumor tissues. A PD-L1 antibody (1:50), a Gal-3 antibody (1:100), a TUNEL antibody (1:100) and a Gal-9 antibody (1:100) were used in IHC. Lastly, diaminobenzidine was used to visualize immunoreactivities. The major organs (heart, liver, spleen, lungs, and kidneys) were paraffin-sectioned, and H&E staining and light microscopy were performed for pathological analysis.

### 4.24 Statistical analysis

Statistical analyses were performed using GraphPad Prism (version 6.0). Data are presented as individual data points with mean ± SD unless otherwise specified in the figure legends. The exact sample size (n) for each experimental group is provided in the corresponding figure legend, representing biological replicates. For comparisons between two groups, a two-tailed unpaired Student's t-test was used. Comparisons among three or more groups were analyzed by one-way or two-way ANOVA, as appropriate, followed by Tukey's post hoc test for multiple comparisons. Survival curves were compared using the log-rank test. All tests were two-sided, and specific P-values are reported in the figures or legends; *P* < 0.05 was considered statistically significant. No formal correction for multiple testing was applied across independent experiments, as each was analyzed separately. In all in vivo studies, including the biodistribution assay, animals were randomly assigned to experimental groups. Whenever possible, investigators were blinded to group allocation during data collection and analysis. In vitro experiments were independently repeated at least three times, and key in vivo findings were confirmed in two independent experiments.

## CRediT authorship contribution statement

**Li Du:** Writing – original draft, Formal analysis, Data curation, Conceptualization. **Xiaoying Zhang:** Writing – original draft, Methodology, Formal analysis, Data curation. **Yao Gong:** Methodology, Investigation, Data curation. **Miaoshu Liu:** Validation, Methodology, Formal analysis. **Jide Sun:** Writing – review & editing, Validation. **Xingping Hu:** Resources, Methodology. **Jian Peng:** Visualization, Supervision, Project administration. **Zhangling Liu:** Supervision, Resources. **Ting Zhang:** Supervision, Software. **Jie Xu:** Validation, Investigation. **Fengxia Gao:** Writing – review & editing, Funding acquisition, Data curation. **Wei Cheng:** Writing – review & editing, Funding acquisition, Conceptualization.

## Declaration of competing interest

The authors declare that they have no known competing financial interests or personal relationships that could have appeared to influence the work reported in this paper.

## Data Availability

Data will be made available on request.
