# Supplementary material for: Activated T-cell membrane-derived nanocargoes displaying multi-immune checkpoints for enhanced cancer immunotherapy
Source: Mater Today Bio. 2025 Dec 24;36:102702. doi: 10.1016/j.mtbio.2025.102702 (PMC12813334; doi:10.1016/j.mtbio.2025.102702)
Supplement: Multimedia component 1 [file mmc1.docx]

Supplementary information

**Activated T-cell membrane-derived nanocargoes displaying multi-immune checkpoints for enhanced cancer immunotherapy**

**Li Du^a,1^, Xiaoying Zhang^a,1^, Yao Gong^a,1^, Miaoshu Liu,^a^ Jide Sun^c^, Xingping Hu^a^, Jian Peng^b^, Zhangling Liu^b^, Ting Zhang^b^, Jie Xu^a^, Fengxia Gao^a,*^, Wei Cheng^a,b,d,*^**

^a^The Center for Clinical Molecular Medical Detection, Innovative and Translational Laboratory of Molecular Diagnostics, Laboratory Medicine Center, the First Affiliated Hospital of Chongqing Medical University, Chongqing 400016, P.R.China.

^b^Biobank, the First Affiliated Hospital of Chongqing Medical University, Chongqing 400016, China.

^c^Department of Laboratory Medicine, the First Affiliated Hospital of Chongqing Medical University, Chongqing 400016, China.

^d^Western Institute of Digital-Intelligent Medicine，Chongqing 401329, China.

*Corresponding authors: gaofengxiayyds@outlook.com (F.G), chengwei@hospital.cqmu.edu.cn (W.C)

^1^These authors contributed equally to this work.


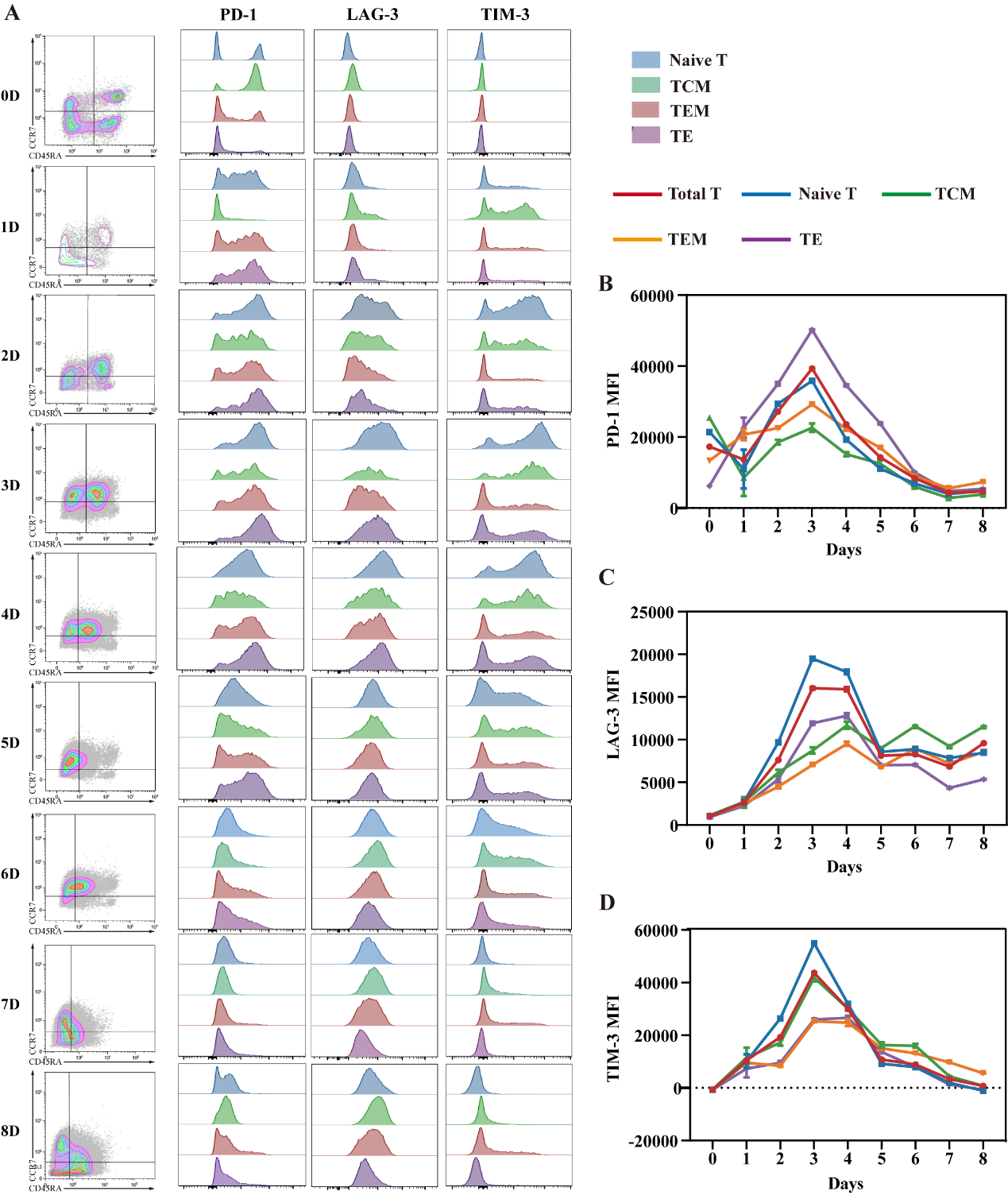


**Fig S1.** Expression of immune checkpoints in different T cell subsets at different stages of activation in vitro.

1. Representative flow cytometric histograms (left) of the expression level of PD-1, LAG-3, TIM-3 of Naïve T cells (CD45RA^+^CCR7^+^), Central memory T Cells (TCM, CD45RA^-^CCR7^+^), Effector memory T Cells (TEM, CD45RA^-^CCR7^-^) and Effector T Cells (TE, CD45RA^+^CCR7^-^) on day 0 to 8 of activation. (B-D) Representative flow cytometric quantification curves of the expression level of PD-1 (B), LAG-3 (C) and TIM-3 (D) of different T cell subsets on day 0 to 8 of activation (n = 3 independent experiments), MFI, mean fluorescence intensity. All data are presented as the mean ± s.d.


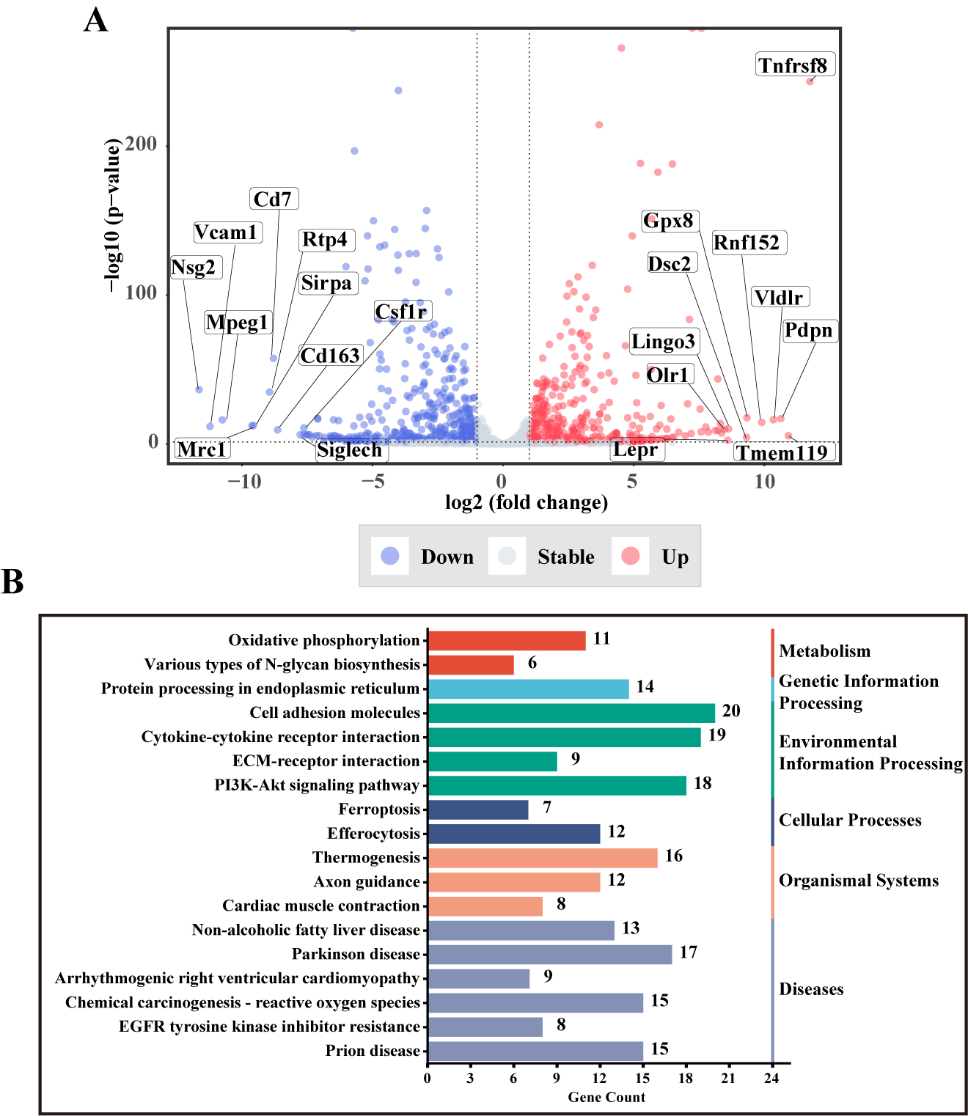


**Fig S2.** Gene expression differences between activated T cells and non-activated T cells.

1. Volcano plot demonstrating the distribution and expression changes of differential genes in the non-activated T cells and specific activated T cells. Genes with absolute fold change >2 and *P* value < 0.05 as highlighted in blue (indicating down-regulated genes) and red (indicating up-regulated genes), respectively. (B) Gene Ontology (GO) enrichment analysis of the up-regulated genes in activated T cells compared with resting T cells.


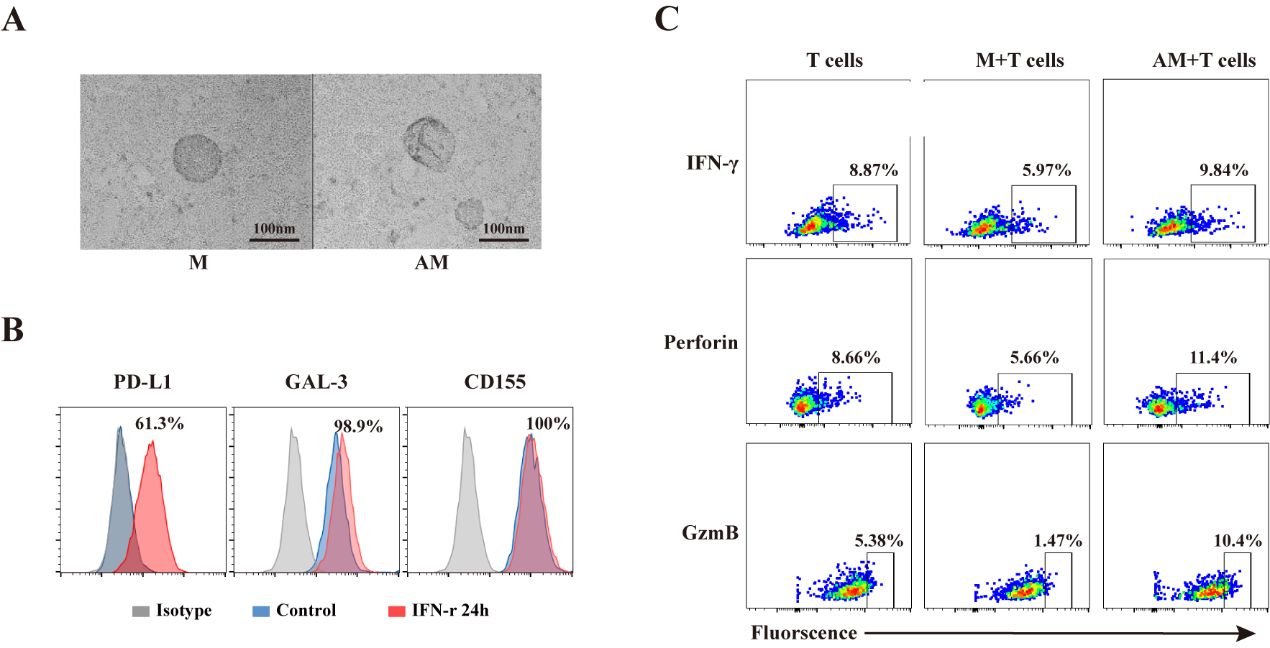


**Fig S3.** The positive rates of PD-L1^+^, GAL-3^+^ and CD155^+^ 4T1-OVA/Luci cells with IFN-γ treatment (20 ng/mL) for 24 h.


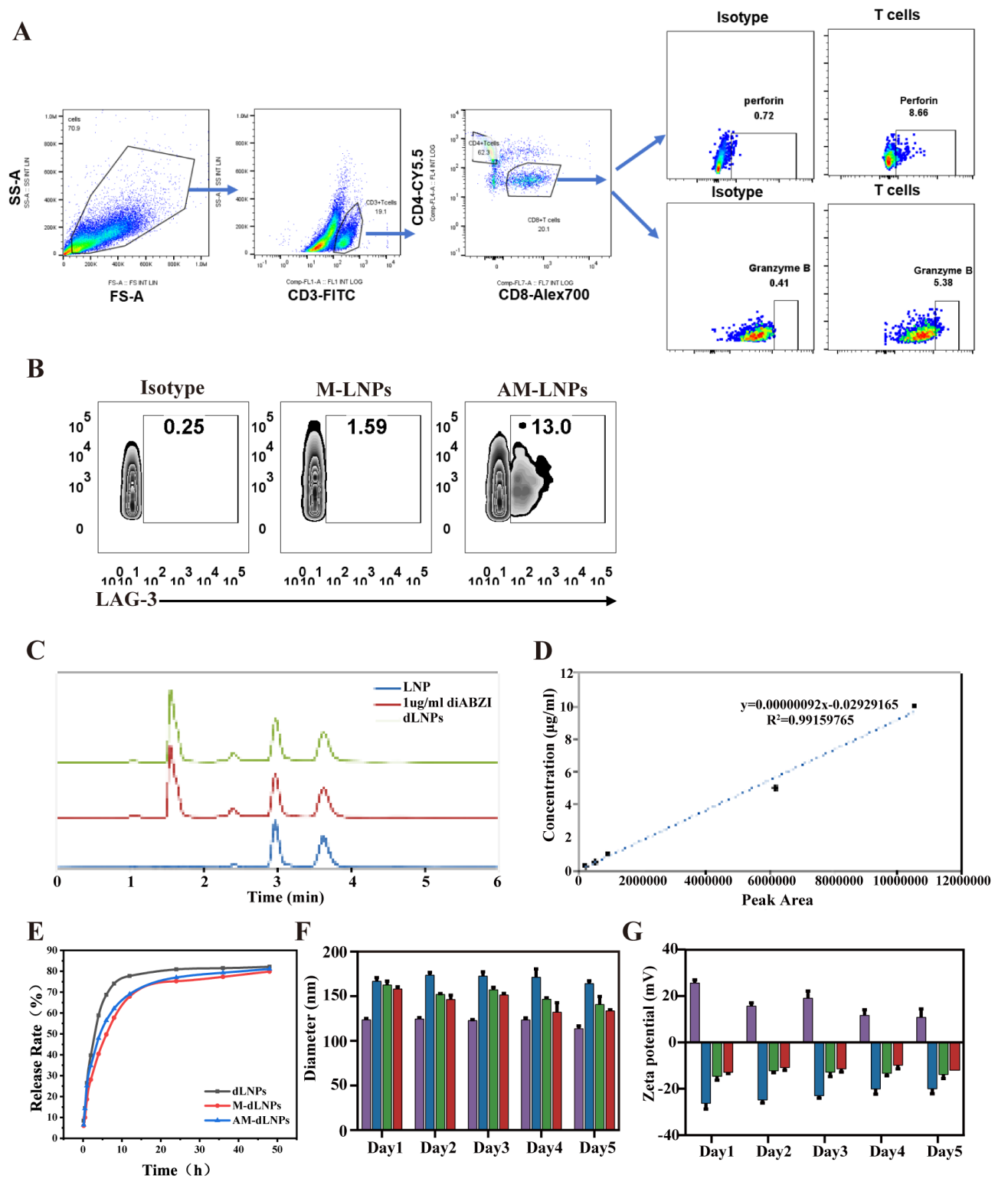


**Fig S4.** Characterization of AM-derived nanocargoes.

1. Representative flow cytometry plots showing the gating strategy for the identification of IFN-γ +, Perforin +, and Granzyme B^+^ populations in OT-1 CD8^+^ T cells. (B) Expression of LAG-3 on AM-derived nanocargoes was determined by Flow NanoAnalyzer. (C) High performance liquid chromatographic (HPLC) peak diagram of LNPs, diABZI and dLNPs. (D) Standard curve of the concentration of diABZI and its corresponding peak area determined by HPLC (n = 3 independent experiments). (E) Leakage rate over time of diABZI from different nanoparticles was determined by HPLC (n = 3 independent experiments). (F, G) Diameter stability (F) and zeta potential stability (G) of different nanoparticles suspended in 50% fetal bovine serum at a nanoparticle concentration of 1 mg/ml were measured by DLS on days1,2,3,4,5 (n = 3 independent experiments). All data are presented as the mean ± s.d.


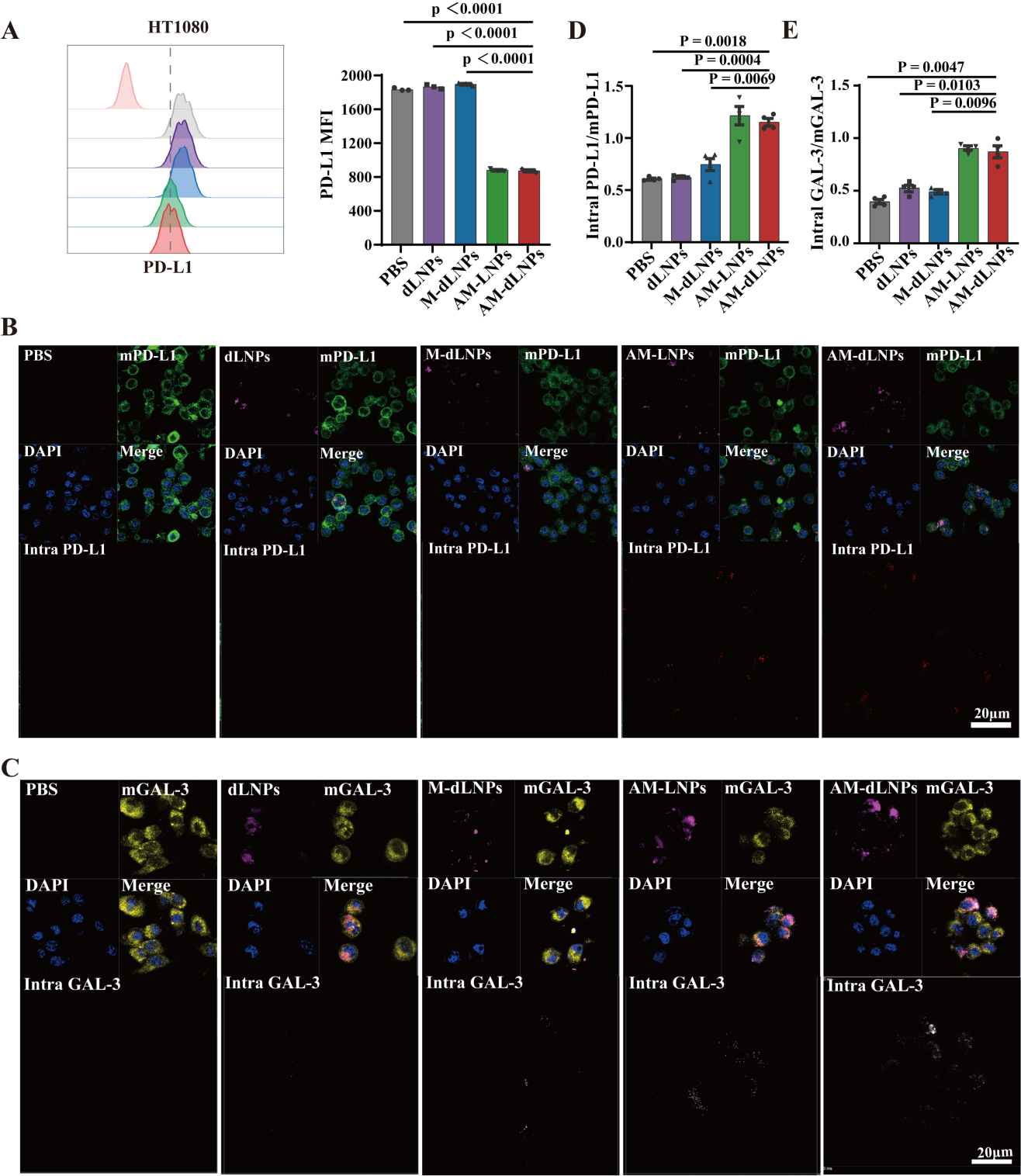


**Fig S5.** AM-dLNPs-mediated PD-L1 binding and internalizing in vitro.

(A) Representative flow cytometric histograms (left) and bar graph (right) of the relative levels of membrane PD-L1 on HT1080 tumor cells after incubation for 4h with dLNPs, M-dLNPs, AM-LNPs and AM-dLNPs or PBS (control) (n = 3 independent experiments). (B-C) Representative confocal images of membrane PD-L1 (mPD-L1), intracellular PD-L1 (Intra PD-L1) (B) and membrane GAL-3 (mGAL-3), intracellular GAL-3 (Intral GAL-3) (C) of IFN-γ-pretreated (20ng/ml, 24h) 4T1 cells after co-incubation with Cy5-labelled dLNPs, M-dLNPs, AM-LNPs and AM-dLNPs (purple) or PBS for 4h. The cells were stained with DAPI (4′,6-diamidino-2phenylindole, blue), membrane anti-PD-L1 antibody (green) and intracellular PD-L1 antibody (red), membrane anti-GAL-3 antibody (yellow) and intracellular GAL-3 antibody (white). Scale bars, 20 µm. (D-E) The ratio of the mean fluorescence intensity of Intra PD-L1 to mPD-L1 (D) and Intra Gal-3 to mGal-3 (E) (n = 3 independent experiments). All the data are presented as the mean ± S.D. The *p* values were determined by one-way ANOVA with Tukey’s post-test for (A) and (D,E).


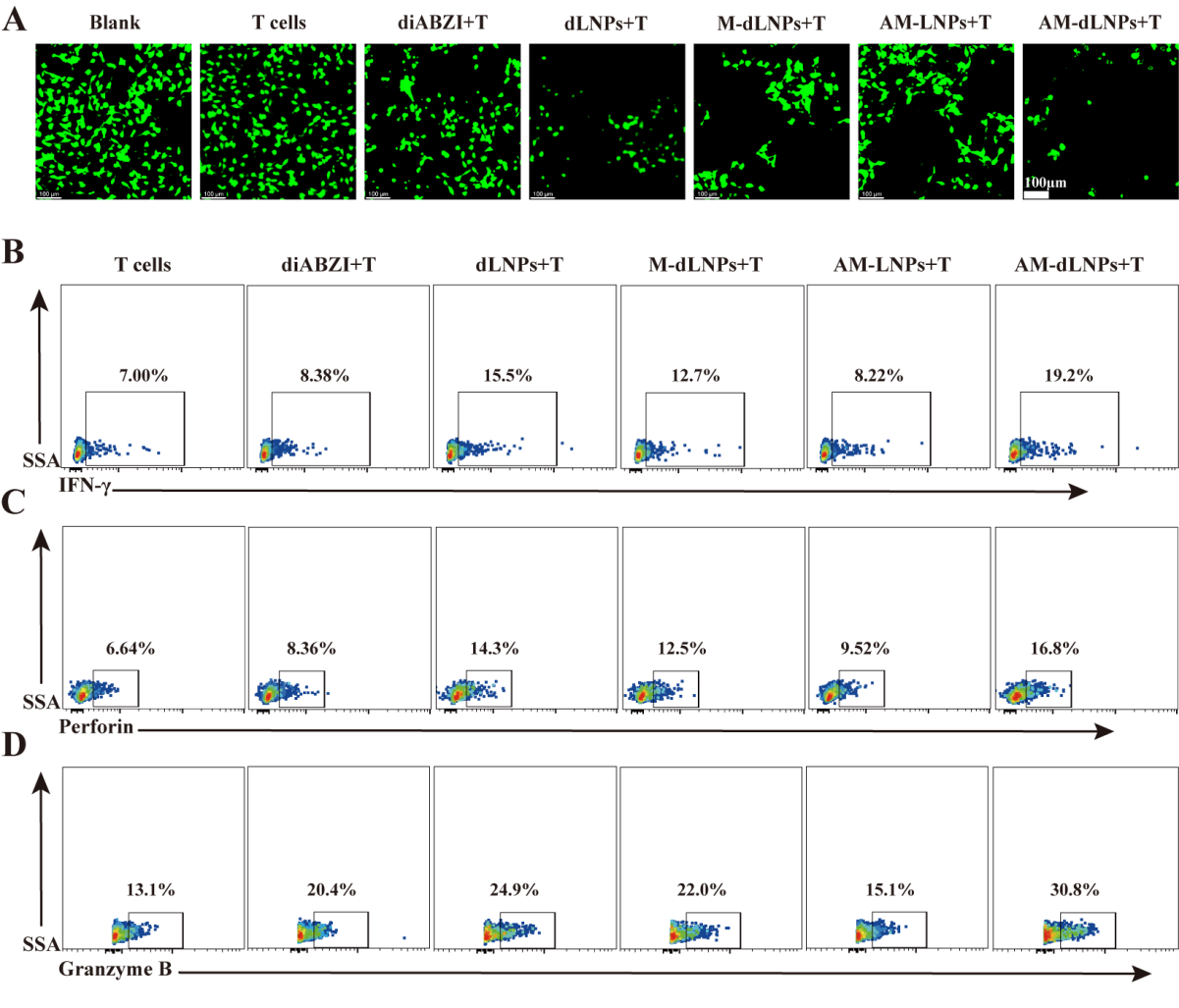


**Fig S6.** Antitumor effects of AM-dLNPs in vitro.

1. The viability of IFN-γ-pretreated 4T1-OVA/Luci cells was detected by Calcein⁃AM staining after co-incubation with OT-1 T cells at an effector: target ratio of 5:1 and diABZI, dLNPs, M-dLNPs, AM-LNPs, and AM-dLNPs or PBS for 24h. Scale bars, 100 µm. (B-D) Representative flow cytometric percentage of IFN-γ^+^ (B), Perforin^+^ (C) and Granzyme B^+^ (D) in aforementioned T cells, gated on CD3^+^ CD8^+^T cells (n = 3 independent experiments).


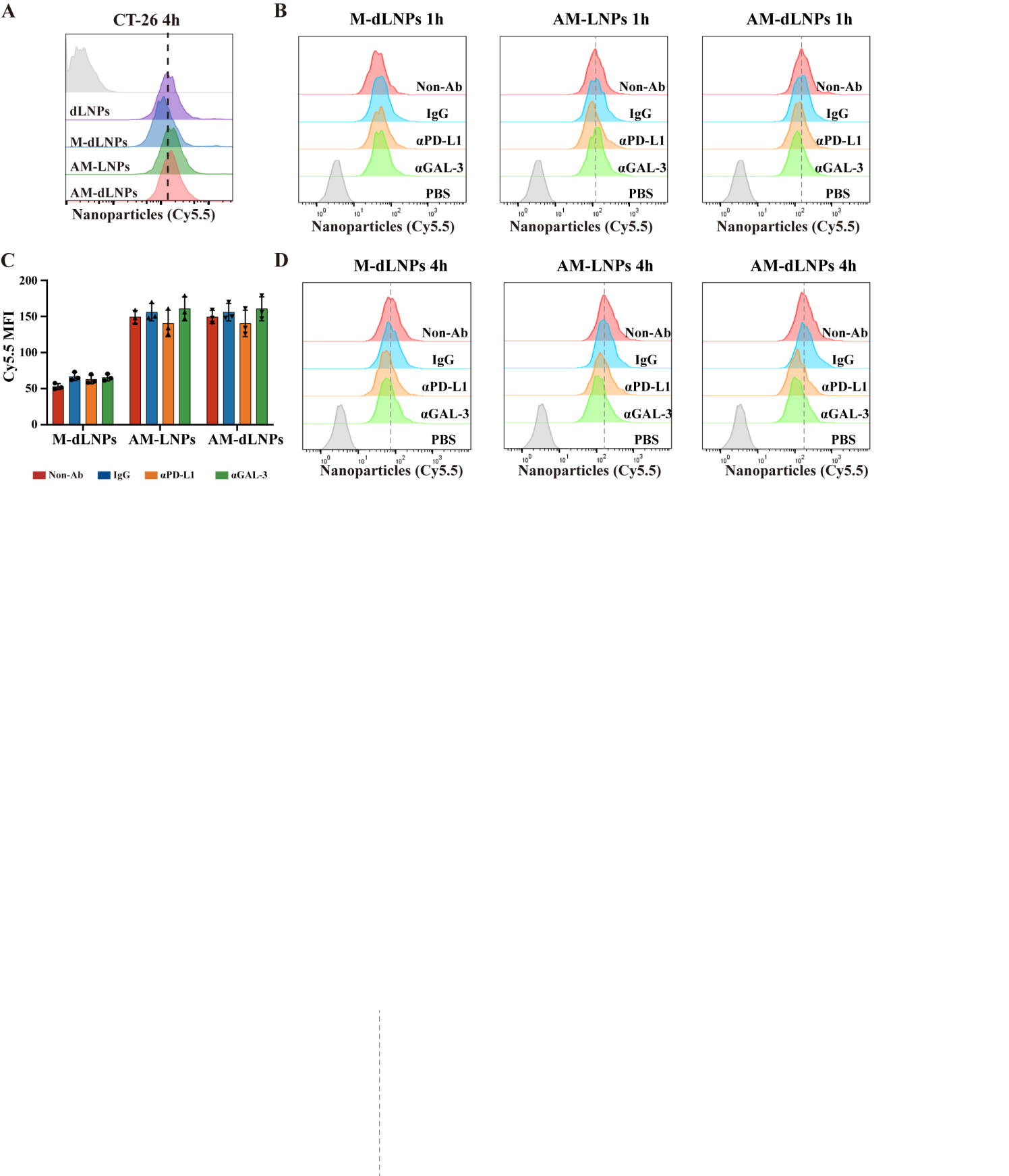


**FigS7.** Cellular uptake of AM-dLNPs in vitro.

(A) Representative flow cytometric histograms of the cellular uptake of nanoparticles by IFN-γ-pretreated CT-26 cells after incubation with different nanoparticles for 4h. The nanoparticles were labelled with Cy5.5. (B-D) Flow cytometric analysis of cellular uptake of nanoparticles by IFN-γ-pretreated CT-26 cells blocked with anti-PD-L1 or anti-GAL-3 antibodies for 2h prior to incubation with nanoparticles for 1h (B-C) and 4h (D). The nanoparticles were labelled with Cy5.5 (n = 3 independent experiments). All the data are presented as the mean ± S.D. The p values were determined by two-way ANOVA with Tukey’s post-test for (C).


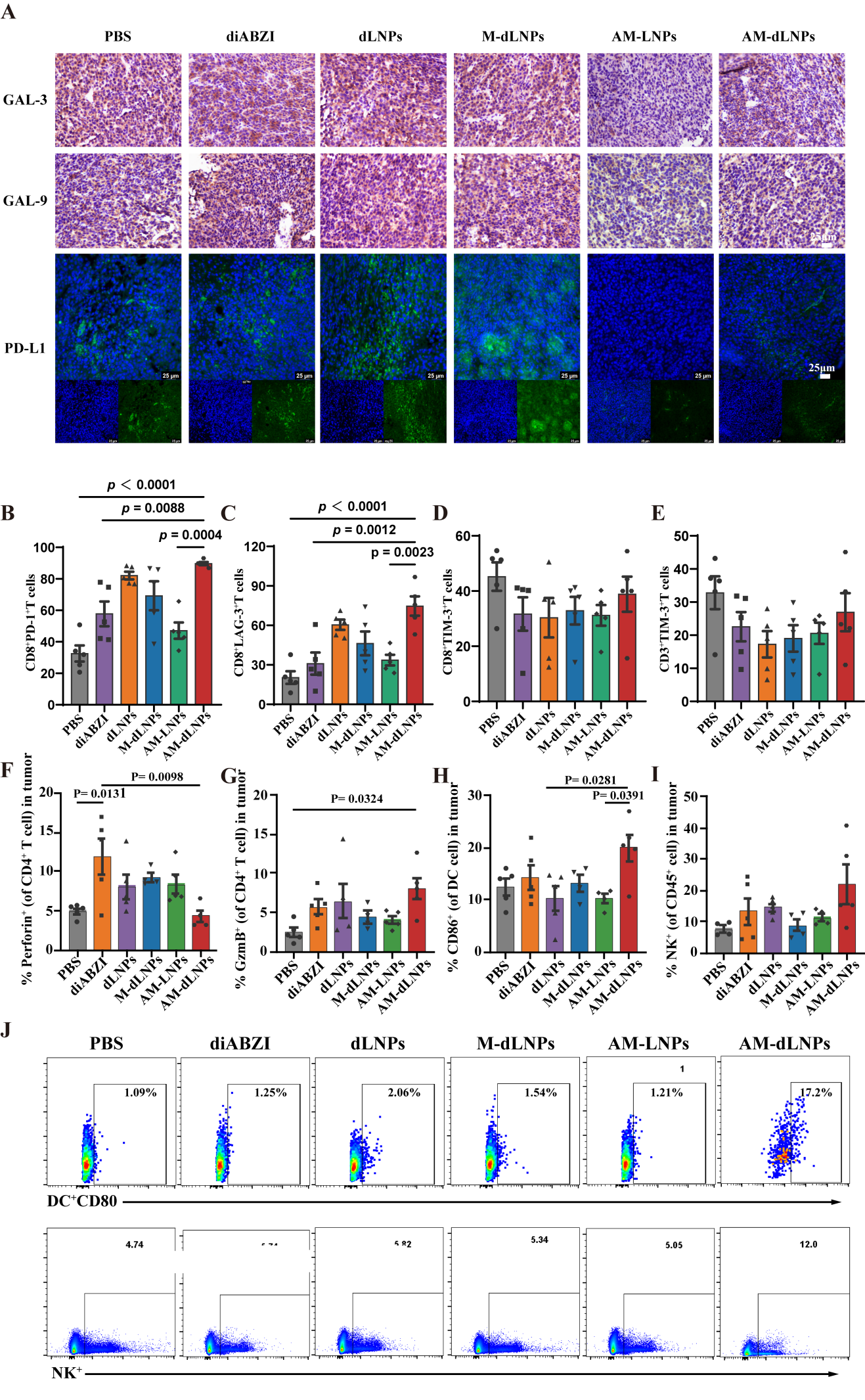


**Fig S8.** AM-dLNPs activated anti-tumor immunity.

(A) Representative IF and immunohistochemistry (IHC) staining of GAL-3 (IHC), GAL-9 (IHC) and PD-L1 (IF) in tumor sections obtained from mice receiving three injections of the indicated treatments. The nuclei were stained with DAPI (blue) in IF. Scale bars, 25 µm (n=5 mice). (B-E) Representative flow cytometry statistical analysis of T cell exhaustion markers PD-1 (A), LAG-3 (B), TIM-3 (C) on CD8^+^T cells and TIM-3 on CD3^+^Tcells across different treatment groups by flow cytometry (n = 5 mice). (F-G) Representative flow cytometric percentage of Perforin (F) and Granzyme B (G) that produced tumor-infiltrating CD4^+^ T cells harvested on day 3 from mice after the last injection of the three-injection regimen (n = 5 mice). (H-I) Representative flow cytometric percentage of CD86^+^ DCs (H) and NK cells (I) in tumors harvested from CT-26-bearing mice on day 3 after the last injection of the three-injection regimen (n = 5 mice). (J) Representative flow cytometric plots of CD80^+^ DCs and NK cells in spleen harvested from CT-26-bearing mice on day 3 after the last injection of the three-injection regimen (n = 5 mice). All the data are presented as the mean ± S.D. The *p* values were determined by one-way ANOVA with Tukey’s post-test for (B-I).


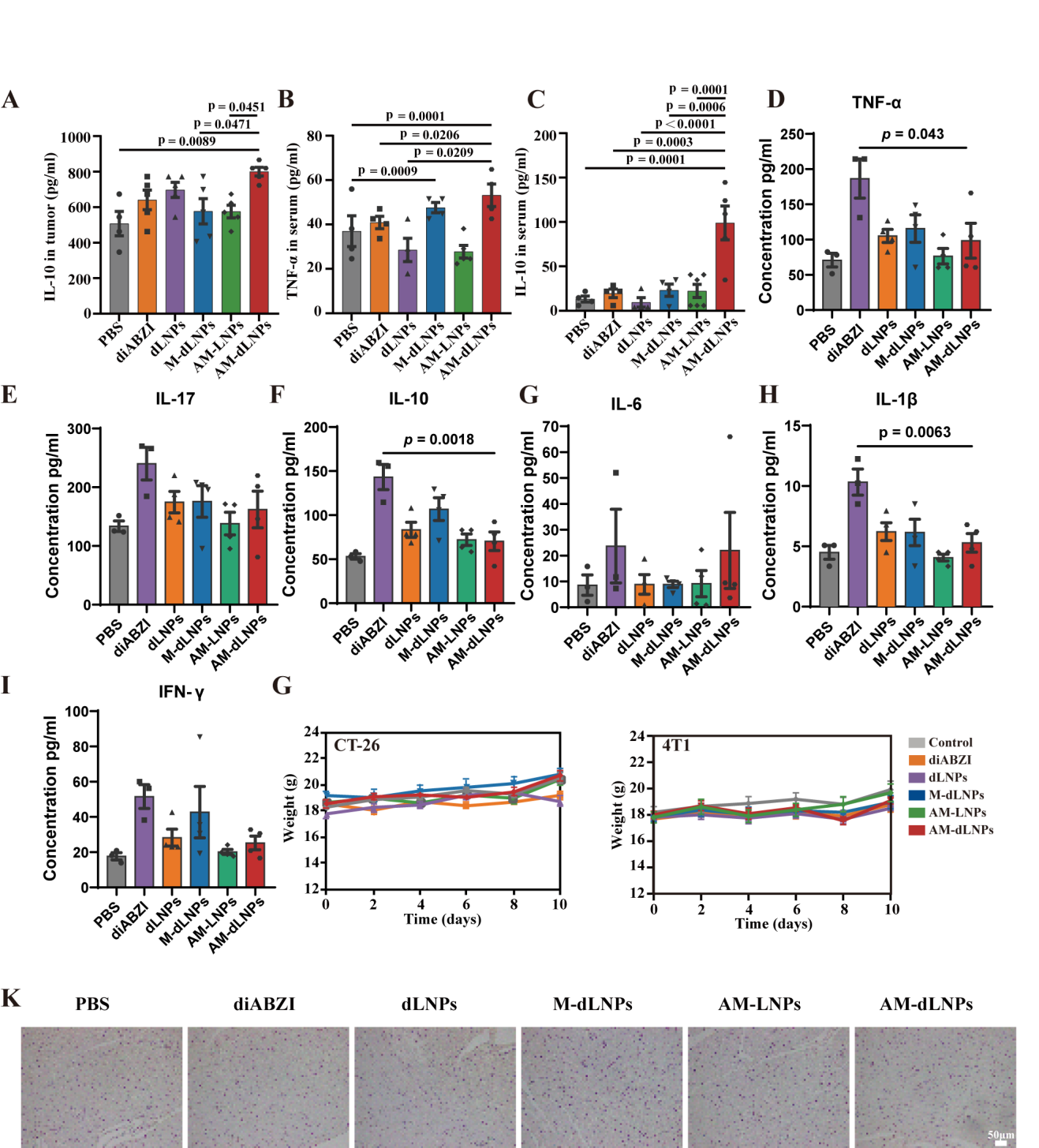


**Fig S9. Biosafety profiles of AM-dLNPs in vivo.**

(A)The quantification of IL-10 in the tumor collected from mice receiving the indicated treatments on day 3 after the third injection (n = 5 mice). (B, C) The quantification of TNF-α (B) and IL-10 (C) in the serum collected from mice receiving the indicated treatments on day 3 after the third injection (n = 4 mice). (D-I) The quantification of TNF-α (D)、IL-17 (E) 、IL-10 (F)、IL-6 (G)、IL-1β (H) and IFN-γ (I) in the serum collected from mice receiving the indicated treatments on day 1after the third injection (n = 3-4 mice). (G) Body weight changes of CT-26 tumor-bearing mice and 4T1 tumor-bearing mice following the indicated treatments (3 injections at day 0, 4, and 8 at a dosage of 0.75 mg/kg diABZI) (n = 5 mice). (K) IHC staining of TUNEL in cardiac tissues obtained from mice receiving three injections of the indicated treatments. Scale bars, 50 µm. All the data are presented as the mean ± S.D. The *p* values were determined by one-way ANOVA with Tukey’s post-test for (A-I); and by two-way ANOVA with Tukey’s post-test for (G).
